# Comparative effectiveness of various physical exercise interventions on executive functions and related symptoms in children and adolescents with attention deficit hyperactivity disorder: A systematic review and network meta-analysis

**DOI:** 10.3389/fpubh.2023.1133727

**Published:** 2023-03-24

**Authors:** Feilong Zhu, Xiaotong Zhu, Xiaoyu Bi, Dongqing Kuang, Boya Liu, Jingyi Zhou, Yiming Yang, Yuanchun Ren

**Affiliations:** ^1^College of Physical Education and Sports, Beijing Normal University, Beijing, China; ^2^School of Social Ecology, University of California, Irvine, Irvine, CA, United States

**Keywords:** attention-deficit/hyperactivity disorder, children and adolescents, network meta-analysis, public health, physical exercise

## Abstract

**Background:**

Physical exercise has been recommended as an important nonpharmacological therapeutic strategy for managing attention deficit hyperactivity disorder (ADHD). We conducted a network meta-analysis (NMA) to assess the comparative impact of different physical exercise modalities on enhancing executive functions (EFs) and alleviating symptoms in children and adolescents with ADHD.

**Methods:**

We searched Web of Science, PubMed, Embase, Cochrane Central Register of Controlled Trials, SPORTDiscus, PsycINFO, CNKI, and clinical trials databases from inception to October 20, 2022. Randomized controlled trials (RCTs) and quasi-experimental studies investigating physical exercise for ADHD-related symptoms of hyperactivity/impulsivity and inattention, and executive functions were included. The frequentist random-effect NMA method was applied to pool the results.

**Results:**

A total of 59 studies (including 39 RCTs, 5 quasi-RCTs, and 15 self-controlled trials) published between 1983 and 2022 were incorporated into the systematic review, of which 44 studies with 1757 participants were eligible for meta-analysis. All types of physical exercise were effective in improving EFs (SMD = 1.15, 95% CI: 0.83 to 1.46), and open-skill activities which require participants to react in a dynamically changing and externally paced environment induced the most incredible benefits for executive functions (SUCRA = 98.0%, SMD = 1.96, and 95% CI: 1.15 to 2.77). Subgroup analyses for EFs revealed varied findings that open-skill activities were the most promising physical exercise type for improving inhibitory control (SUCRA = 99.1%, SMD = 1.94, and 95% CI: 1.24 to 2.64), and closed-skill activities dominated by aerobic exercises had a slightly higher probability of being the most promising physical exercise intervention for working memory (SUCRA = 75.9%, SMD = 1.21, and 95% CI: −0.22 to 2.65), and multicomponent physical exercise tended to be the most effective in cognitive flexibility (SUCRA = 70.3%, SMD = 1.44, and 95% CI: −0.19 to 3.07). Regarding ADHD-related symptoms, closed-skill activities dominated by aerobic exercises might be more advantageous for hyperactivity/impulsivity (SUCRA = 72.5%, SMD = -1.60, and 95% CI: −3.02 to −0.19) and inattention (SUCRA = 96.3%, SMD = -1.51, and 95% CI: −2.33 to −0.69) improvement.

**Conclusion:**

Physical exercise can significantly help to alleviate the symptoms of ADHD and improve executive functions in children and adolescents with ADHD. Most of all, to promote adherence to treatment, they should be encouraged to perform the physical exercises that they enjoy most.

## 1. Introduction

With a global prevalence of 5.29 to 7.2%, attention-deficit/hyperactivity disorder (ADHD) is the most common neurodevelopmental psychiatric disorder in childhood and generally persists into adulthood (1). ADHD is generally characterized by developmentally inappropriate levels of “core symptoms,” including inattention, hyperactivity and impulsivity (2), which affect children’s behavior, emotion, and executive functions (EFs) and may lead to multidimensional difficulties in their academic and social abilities (3–6).

The symptoms of ADHD can be categorized into two types of behavioral problems (2). Inattention is a prominent symptom of ADHD and a necessary symptom for diagnosis. Children with ADHD are characterized by abnormalities in concentration, stability, and selectivity of attention, lacking resistance to extraneous stimuli. Hyperactivity/impulsivity is another major symptom of ADHD, manifested by a significantly higher level of activity than typically developing children, which is more pronounced in situations requiring sitting down or the need for order.

Executive function deficits are a hallmark characteristic of individuals with ADHD. Executive functions refer to advanced cognitive skills that assist with planning, organizing, problem solving and managing, and they contain three core components: inhibitory control, working memory, and cognitive flexibility (7, 8). According to Barkley’s response inhibition model theory, impairment of inhibitory function is the primary cause of ADHD symptoms and secondary impairment of executive function (9). Numerous studies have reported that children and adolescents with ADHD have lower reaction speeds and correctness scores than control subjects with typical development (10, 11) and have verified that children with ADHD struggle greatly with EFs, including managing impulsive behavior, cognition, and attention; they routinely make careless mistakes while doing schoolwork and have difficulty paying attention when performing tasks or playing (12).

Currently, medications and behavioral and psychological therapy are the mainstays of ADHD treatment (13, 14). Researchers have found that more than one-third of children do not respond, respond only partially, or encounter adverse drug reactions to these medications (15), and it may be challenging to adhere to these drug regimens owing to their time-consuming nature and high financial cost (16). In recent years, physical exercise interventions for children and adolescents with ADHD have gained increased attention from researchers. Several studies have concluded that 6 weeks of football exercise helped to improve the EFs of boys with ADHD (17), and an 8-week swimming program enhanced mental health and cognitive and motor coordination parameters in children with ADHD (18). There were also meta-analysis studies showing that exercise significantly affected children and adolescents with ADHD (16, 19); physical exercise helps them to engage in meaningful sensorimotor interactions with their surroundings and develop the ability to structure their brain activity patterns, which are crucial for executive functions (20). Physical exercise also has the potential to act as an endogenous stimulus to set off a series of neuroplastic molecular processes that eventually result in structural adaptations and changes in the network activity of the brain, thereby enhancing neurotransmitter systems and upregulating brain-derived neurotrophic factor (BDNF) and neurogenesis (20). Additionally, by boosting dopamine and norepinephrine neurotransmitters, physical exercise may act physiologically similarly to stimulant drugs for alleviating the symptoms of ADHD. In combining the above results, physical exercise has been recommended as an important nonpharmacological therapeutic strategy for managing ADHD.

Traditional meta-analysis approaches fail to answer important questions about which form of intervention works the best, and no previous systematic studies have reported which physical exercise is optimal for enhancing executive functions and alleviating symptoms in children and adolescents with ADHD. This is a complicated question that deserves more research and has important implications for public health and education. Network meta-analysis (NMA) is a novel analytic approach combining direct and indirect evidence that allows a comparison of the effects of more than two interventions simultaneously in a single analysis. Importantly, it enables alternative interventions to be ranked for a single outcome and displays the probability of each intervention’s relative efficacy, which can be helpful in guiding therapeutic decision-making (21, 22). We performed a systematic review and network meta-analysis that compared the relative efficacy of different modalities of physical exercise based on direct and indirect evidence and aimed to identify the optimal physical exercise intervention for improving executive functions and alleviating major symptoms in children and adolescents with ADHD.

## 2. Methods

### 2.1. Protocol

This study was conducted in accordance with the Preferred Reporting Items for Systematic Reviews and Meta-Analyses for Network Meta-Analyses (PRISMA-NMA) (23), and the PRISMA checklist is provided in Appendix 1. We registered our study in the PROSPERO platform under registration number CRD 42022365188, and we followed the protocol for all the steps of this systematic review and network meta-analysis.

### 2.2. Search strategy

The search strategy was developed, piloted, and refined according to the Patient population, Intervention, Comparison, Outcome (PICO) approach and previously published systematic reviews (16, 19, 24). We performed the literature search in several English-language databases, including Web of Science, PubMed, Embase, Cochrane Central Register of Controlled Trials, SPORTDiscus, PsycINFO and clinical trials.^1^ The authors also searched the Chinese-language database (CNKI) using the same terms in Chinese following the updated study protocol. The updated study protocol and protocol deviations are given in Appendix 2 S1 Table S1. We limited publication dates from inception to October 20, 2022, and no language restrictions were applied. We also hand-searched reference lists of included studies and related systematic reviews to ensure complete capture. The details of the search strategy are presented in Appendix 2 S2.

### 2.3. Inclusion and exclusion criteria

The criteria for inclusion were as follows: (a) participants aged <18 years (children or adolescents) with a diagnosis of ADHD of any subtype; (b) interventions involving the physical exercise program with no limitation on the frequency, intensity, time, and types; (c) the comparators were controlled (e.g., waiting list, no intervention, watching video, and sedentary attention control); (d) the outcomes were ADHD-related symptoms of hyperactivity/impulsivity and inattention, and executive functions as measured using several scales and tests; (e) RCTs, quasi-RCTs (participants were allocated to groups using nonrandom methods, e.g., consecutive appearance, day of the week), and self-controlled trials were eligible. (f) The studies were included regardless of medication status. If medicine was used as a comparative intervention in the control group, the study will be solely used as a qualitative review rather than a quantitative meta-analysis. Studies were excluded if they met the following criteria: (a) interventions that did not involve physical exercise or (b) noninterventional clinical trials, such as protocols, review studies, cohort studies, case–control studies, conference papers, and book chapters.

### 2.4. Study selection and data extraction

The titles and abstracts of the studies found by using the search strategy were independently reviewed by two authors. The final decision was then made after reading the full texts of the remaining studies. The interrater agreement for the authors’ abstract and full text screening was calculated as the Kappa score by IBM SPSS Statistics 25.0 (SPSS, Chicago, IL, United States), which was judged as excellent (≥0.75), good (0.60–0.74), fair (0.40–0.59), and poor (<0.40).

We did not restrict the studies by language and translation where feasible, and we manually omitted studies featuring obvious errors. Two authors separately extracted the data, and any discrepancies were discussed among the authors to come to a conclusion. If a disagreement persisted, the outcome was decided by a third author. The study characteristics (first author’s name, publication year, country, and study design), participant characteristics (sample size, age, and sex ratio), intervention characteristics (type, frequency, intensity, duration per session, and length of intervention), comparator information, and outcomes (the pertinent statistics at the intervention’s endpoint for estimating effect sizes) were all presented in the data extraction table. The mean in each group was multiplied by −1 when the studies were reverse-scaled (greater values indicated lower outcomes rather than better results). Where standard deviations (SDs) were not provided, they were derived from standard errors (SEs), confidence intervals (CIs), or t or *p* values, or attempts were made to contact the authors at least three times through e-mails to collect the missing data. GetData Digitizer version 2.20 software was used to extract the data we required from graphs when the authors did not provide the data in the study but instead provided a graph containing the data.

### 2.5. Data coding and management

The physical exercise interventions were classified according to following four hierarchical levels, which was more suitable for children and adolescents and agreed with the research steering committee. First, the interventions were coded as “Physical exercise” or “Control.” At the second level, the interventions were coded based on their main characteristics: “Sports,” “Aerobic exercise,” “Mind–body exercise,” “Mixed physical exercise,” “Exergaming,” “Specific training modality.” At the third level, the interventions were coded according to the specific type of exercise performed: “Football,” “Table tennis,” “Badminton,” “Tennis,” “Swimming,” “Cycle ergometer,” “Running,” “Yoga” etc. Lastly, interventions were coded at the intersection of a specific type with the following broad categories: open-skill activities (require participants to react in a dynamically changing and externally paced environment and promote the use of the brain while performing the task, such as football, table tennis, badminton, tennis); closed-skill activities (which require participants to perform in a highly consistent, stationary, and self-paced environment, such as swimming, running, cycle ergometer, rope skipping); multicomponent physical exercise (the combination of open-skill and closed-skill activities, such as swimming and racket sports); exergaming (the combination of physical and cognitive training in a gamified fashion); and specific training technique that do not target any particular movement (high-intensity interval training, HIIT; moderate-intensity continuous training, MICT). For the intensity of the physical exercise, we defined low intensity (<60% HRmax/45% VO2max), moderate intensity (60–75% HRmax/45–65% VO2max), and vigorous intensity (>75% HRmax/65% VO2max).

### 2.6. Study quality assessment and quality of evidence

The methodological quality of the included RCTs and quasi-RCTs was assessed by two authors using the Physiotherapy Evidence Database scale (PEDro) (25). Disagreements were discussed between the authors to reach a consensus. The PEDro scale contained 11 items, which included the eligibility criteria, random allocation, concealed allocation, baseline comparability, blind subjects, blind therapists, blind assessors, adequate follow-up, intention-to-treat analysis, and between-group comparisons as well as point estimates and variability. The minimum score was 0 points, and the maximum score was 10 points. The score was categorized as follows: <4 points as poor quality, 4 to 5 points as fair quality, 6 to 8 points as good quality, and 9 to 10 points as excellent quality. The Joanna Briggs Institute (JBI) Critical Appraisal Tools was used to assess the quality and risk of bias of self-control trials (26). The tool consists of 9 questions whose answers could be “yes,” “no,” “unclear,” and “not applicated.”

The Grading of Recommendations, Assessment, Development and Evaluations (GRADE) system was used in this study to judge the quality of evidence (27). Based on the GRADE’s five downgraded criteria with reference to the risk of bias, inconsistency, indirectness, imprecision, and publication bias, we determined whether to downgrade the quality level of the evidence. Ultimately, the certainty of the evidence was rated as high, moderate, low, and very low.

### 2.7. Statistical analysis

It was determined with the research steering committee that self-controlled trials would be included to capture data on emerging treatments and treatments tested in more pragmatic settings. If the included studies were self-controlled trials (pre-intervention vs. post-intervention in one individual), we used appropriate descriptive analyses to describe the individual and summarized the characteristics of these studies. Due to recruitment difficulties in a few studies, the authors used a quasi-RCT design, which participants were allocated to groups using nonrandom methods, and participants might have been recruited from the same school as one group. If randomized and nonrandomized exercise studies produced homogeneous effect size estimates, one could pool the results (28). Eventually, with the included studies being RCTs and quasi-RCT (without randomization but with baseline comparability), we performed a meta-analysis to evaluate the efficacy quantitatively.

First, a pairwise meta-analysis was performed across all outcomes to investigate the effects of various physical exercise interventions compared to the control group. For the conventional meta-analysis, the heterogeneity across studies was quantified using the *I*^2^ statistic, in which values of 25, 50, and 75% indicated mild, moderate, and high heterogeneity, respectively (29). When the heterogeneity was considerable (*I*^2^ ≥ 50%), a random-effects model was used. Forest plots were generated using R-evolution version 4.2.1.

The network meta-analysis with the frequentist model was performed by combining direct and indirect evidence from all the available studies. We used STATA 17.0 (Stata, College Station, TX, United States.) software within the ‘Network’ package to perform a multivariate random-effects meta-analysis, which can account for heterogeneity caused by clinical and other factors across studies and provide a more conservative confidence interval for pooled point estimates. The graphs of the network plot were presented, which showed the comparative relationships among the groups. Effect sizes were estimated with the standardized mean difference (SMD) and 95% confidence intervals (95% CIs) using means and standard deviations of postintervention scores. According to the SMD statistic, Cohen’s categories were used to determine the magnitude of the effect size, with scores of d ≥ |0.8| being considered a large effect, ≥|0.5| to <|0.8| as medium, ≥|0.2| to <|0.5| as small and <|0.2| as trivial (30).

We assessed the network transitivity by comparing the clinical and methodological features to guarantee that multiple treatment comparisons were sufficiently similar. Inconsistency and consistency models were tested via the design by treatment interaction model (i.e., global approach) and node-splitting test (i.e., local approach), which determines the significant difference between direct and indirect comparisons for each treatment with the aim of assessing the consistency. Probability values were summarized and reported as the surface under the cumulative ranking (SUCRA) curve. The SUCRA value would be 0 when a treatment is certain to be the worst and 1 when it is certain to be the best. To evaluate the stability of the results, further sensitivity analyses were conducted by (1) excluding trials judged as poor and fair quality, (2) evaluating only RCTs and (3) being only written in English language. Subgroup analyses according to the different core components of executive functions were conducted. Moreover, we also conducted a meta-regression analysis (with the year of study, study design, age, medication use, frequency, intensity, duration, and length of physical exercise as covariates) for the outcomes. Publication bias and small-study effects were detected through an asymmetrical funnel plot.

Since there were many indirect comparisons in the network meta-analysis, which may raise challenges in reaching appropriate conclusions, we used a partially contextualized framework to draw conclusions from a network meta-analysis according to published GRADE guidance (31). First, we chose the intervention most connected to other interventions in the network and used that intervention as a reference (e.g., Controls). Second, we used the point estimate by comparing each of the interventions against the reference. Whether the confidence interval crosses the null would be irrelevant, because the NMA is more likely to have wide and imprecise estimates. Third, we used the GRADE system to judge the certainty of the evidence for every intervention compared with the reference. Fourth, more importantly, we checked the consistency between point estimates and SUCRA values. For example, an intervention with a large effect ranks higher than an intervention with a moderate effect; however, the first intervention has a considerably lower SUCRA value than the second intervention, which may be problematic.

## 3. Results

### 3.1. Descriptions of included studies

**Figure 1:**
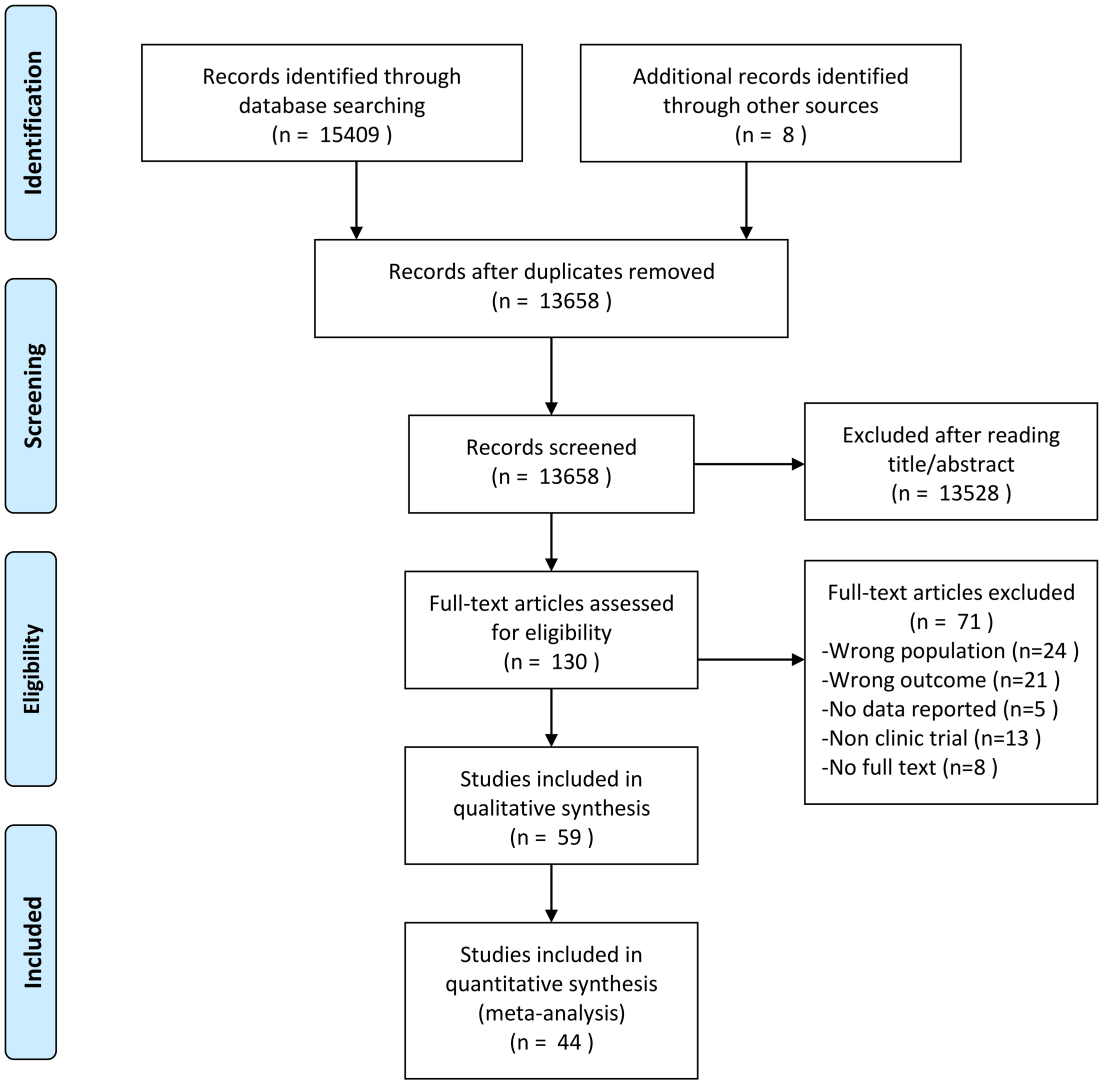
PRISMA flow diagram of the search process for studies.

We identified 15,409 potentially eligible studies through the initial electronic database search and 8 studies through reference list checking in a manual search. Based on a screening of the title and abstract, 13,528 articles were discarded, and we considered 130 potentially eligible studies for inclusion and retrieved their full-text articles. Following application of the inclusion and exclusion criteria, 59 studies (published between 1983 and 2022) including 39 RCTs, 5 quasi-RCTs, and 15 self-controlled trials were selected for this systematic review. The PRISMA-NMA flow chart for study literature selection is given in Figure 1. The agreement rates for study selection by reading the title and abstract and full-text review between two authors were 0.77 and 0.87, respectively (Appendix 2 S3 Tables S2, S3). Of the included studies, 23 examined the effects of closed-skill activities with a focus on aerobic exercises (e.g., swimming, running, and cycle ergometer), 19 examined the effects of multicomponent exercise, 12 examined the effects of open-skill activities (e.g., racket sports, football, and equestrian), 3 examined the effects of exergaming exercise, and 2 examined the effects of HIIT. In total, 44 studies (26 studies for executive functions, 11 studies for symptoms, and 7 studies for both) with 1757 participants were selected to perform the network meta-analysis quantitatively. The median length of therapy was 12 weeks, and each session lasted 45 min. The physical exercise intensity was mostly moderate or moderate-to-vigorous. Regarding regions, 20 studies were conducted in East Asia, 12 in North America, 10 in Europe, and 7 in Africa; the remaining studies were conducted in other regions. A total of 8 studies did not report the participants’ sex, and the ratio of male to female was about 4:1. The characteristics of the studies included in this systematic review are presented in Table 1.

**Table 1:** Characteristics of studies included in the review.

Study	Country	Study design	N	Sex (M/F)	Age (year)	Physical activity					Control arm	Outcomes	Medication use
						Type	Intensity	Duration (min)	Frequency (times/week)	Week			
Ziereis et al. (2015) (32)	Germany	RCT	43	32/11	7–12	Ball handling, balance and manual dexterity; Sports	NR	60	1/week	12	Waiting-list	Executive function	None
Benzing et al. (2019) (33)	Switzerland	RCT	51	43/8	8–12	Exergaming	Moderate	30	3/week	8	Waiting-list	Executive function; Core symptom	NR
Messler et al. (2018) (34)	Germany	RCT	28	28/0	8–13	HIIT	Vigorous	60	3/week	3	Standard multimodal therapy	Core symptom	Yes
Kang et al. (2011) (35)	Korea	RCT	32	32/0	7–10	Aerobic, goal-directed exercise, motor skill	NR	90	2/week	6	Education	Executive function; Core symptom	Yes
Bustamante et al. (2016) (36)	USA	RCT	35	24/11	6–12	Structured activity	Moderate	15	5/week	10	Sedentary attention control	Executive function	Yes
Chang et al. (2012) (37)	Taiwan	RCT	40	37/3	8–13	Treadmill	Moderate	30	1/week	1	Watching video	Executive function	NR
Chang et al. (2014) (38)	Taiwan	RCT	27	23/4	5–10	Aerobic exercise aquatic, motor skill, coordination, balance, and power	Moderate	90	2/week	8	Waiting-list	Executive function	NR
Choi et al. (2015) (39)	Korea	RCT	35	NR	13–18	Running	Moderate	90	3/week	6	Education	Executive function; Core symptom	Yes
Kadri et al. (2019) (40)	Tunisia	RCT	40	36/4	11–18	Taekwondo	Moderate	50	2/week	72	Regular standard multimodal therapy	Executive function	No
Chang et al. (2022) (41)	Taiwan	RCT	48	39/9	7–10	Table tennis; Exergaming	NR	60	3/week	12	No intervention	Executive function	Yes
Chuang et al. (2015) (42)	Taiwan	RCT	19	16/3	8–12	Aquatic exercise	MVPA	30	1/week	1	Watching viedo	Executive function	None
Pan et al. (2016) (43)	Taiwan	RCT	32	32/0	6–12	Table tennis	NA	70	2/week	12	No intervention	Executive function	Yes
Memarmog-haddam et al. (2016) (44)	Iran	RCT	40	40/0	7–11	Selected exercise program, aerobic and goal-directed exercise; table tennis racket and balls	MVPA	90	3/week	8	No intervention	Executive function	None
Smith et al. (2019) (45)	United States	RCT	29	15/14	5–9	Multi-faceted treatment	NR	15	3/week	15	Treatment as usual	Executive function	Yes
Ahmed et al. (2011) (46)	Egypt	RCT	84	54/30	11–16	Aerobic exercise	Moderate	40–50	3/week	10	No intervention	Core symptom	NR
Hattabi et al. (2019) (47)	Tunisia	RCT	40	NR	9–12	Coordination, balance, and power to reinforce different aspects of motor skills	Moderate	90	3/week	12	No intervention	Executive function	None
Hoza et al. (2014) (48)	United States	RCT	104	58/46	5–9	Aerobic physical activity	MVPA	31	7/week	12	Sedentary classroom intervention	Core symptom	Yes
Jensen et al. (2004) (49)	Australia	RCT	19	19/0	8–13	Yoga	NR	60	1/week	20	Talking and listening, cooperative activities	Core symptom	Yes
Garcia-Gomez et al. (2016) (50)	Spain	RCT	18	12/6	7–14	Equestrian	NR	NR	2/week	12	Waiting-list	Core symptom	Yes
Porter et al. (1984) (51)	United States	RCT	34	34/0	NR	Relaxation exercise	Moderate	25	1/week	3	Listening to the story	Executive function; Core symptom	None
Silva et al. (2020) (18)	Brazil	RCT	20	14/6	11–14	Swimming	NR	45	2/week	8	No intervention	Executive function	NR
Oh et al. (2018) (52)	Korea	RCT	34	31/3	6–12	Horseback riding	NR	60	2/week	12	Medication	Core Symptom	Yes
Liu et al. (2018) (53)	China	RCT	64	32/32	7–13	Multicomponent exercise	Moderate	35	3/week	14	No intervention	Executive function	None
Bahram et al. (2014) (54)	Iran	RCT	30	0/30	9.47 ± 1.98	Running	Moderate	20–35	3/week	12	No intervention	Core symptom	None
Hattabi et al. (2021) (55)	Tunisia	RCT	40	NR	Mean = 12	Plyometric training program	Vigorous	30	2/week	12	No intervention	Executive function	None
Felmet et al. (1998) (56)	United States	RCT	40	40/0	9.7 ± 1.36	Karate	MVPA	45	2-3/week	8	Waiting-list	Core symptom	None
Gelade et al. (2018) (57)	Netherlands	RCT	59	46/13	7–13	Aerobic exercise	MVPA	45	3/week	10–12	Medication	Executive function; Core symptom	None
Gelade et al. (2017) (58)	Netherlands	RCT	73	55/18	7–13	Aerobic exercise	MVPA	45	3/week	10–12	Medication	Executive function	None
Soori et al. (2020) (59)	Iran	RCT	43	20/23	12.53 ± 0.3	HIIT	Vigorous	NR	3/week	6	No intervention	Core symptom	None
Song et al. (2022) (17)	China	RCT	16	16/0	6–9	Football	NR	60	5/week	6	Waiting-list	Executive function	None
Chen et al. (2022) (60)	China	RCT	64	53/11	6–10	Cycling on an ergometer	MVPA	25	3/week	12	Watching Video	Executive function; Core symptom	None
Xu et al. (2021) (61)	China	RCT	40	29/11	4–6	Combination exercise	NR	45	2/week	24	No intervention	Core symptom	None
Rezaei et al. (2018) (62)	Iran	RCT	14	NR	7–11	Yoga	NR	45	3/week	8	No intervention	Executive function	Yes
Benzing et al. (2018) (63)	Switzerland	RCT	46	38/8	8–12	Exergaming	moderate	15	1/week	1	Watching Video	Executive function	Yes
Lee et al. (2017) (64)	Korea	RCT	12	12/0	7–9	Multi-sports exercise	Moderate	60	3/week	12	No intervention	Executive function	None
Gawrilow et al. (2013) (65)	Germany	RCT	38	18/20	8–13	Trampoline	vigorous	5	1/week	1	Sedentary	Executive function	NR
Faramarzi et al. (2016) (66)	Iran	RCT	20	20/0	8.60 ± 0.82	Multicomponent exercise	Moderate	45	2/week	6	No intervention	Executive function	None
Smith et al. (2020) (67)	United States	RCT	80	53/27	5–9	Agility ladder, ball skill acquisition, hula-hoop, juggling	NR	45	3/week	15	Treatment as usual	Executive function; Core symptom	Yes
Liang et al. (2022) (68)	Hong Kong	RCT	80	62/18	6–12	Combined exercise	MVPA	60	3/week	12	Waiting-list	Executive function	None
Pan et al. (2019) (69)	Taiwan	quasi-RCT	30	30/0	7–12	Table tennis	Moderate	70	2/week	12	No intervention	Executive function	Yes
Verret et al. (2012) (70)	Canada	quasi-RCT	21	NR	7–12	Aerobic, muscular, and motor skill	MVPA	45	3/week	10	No intervention	Executive function	Yes
Ludyga et al. (2020) (71)	Switzerland	quasi-RCT	18	NR	11–16	Cycling on an ergometer	Moderate	20	1/week	1	Watching video	Executive function	Yes
Chou et al. (2017) (72)	Taiwan	quasi-RCT	49	38/11	8–12	Yoga	Moderate	40	2/week	8	No intervention	Executive function	None
Silva et al. (2015) (73)	Brazil	quasi-RCT	28	NR	10–16	Exergaming	Vigorous	5	1/week	1	No intervention	Core symptom	None
So et al. (2017) (74)	Korea	Self-control	10	NR	10–12	Horseback riding	NR	40	2/week	4	---	Core Symptom	NR
Hernandez-Reif et al. (2001) (75)	United States	Self-control	13	11/2	13–16	Tai Chi	NR	30	2/week	5	---	Core symptom	NR
Lufi et al. (2011) (76)	Israel	Self-control	15	15/0	8–14	Multi-sports activities	NR	75–110	1/week	20	---	Core symptom	None
Cuypers et al. (2011) (77)	Canada	Self-control	5	5/0	10–11	Horseback Riding	NR	60	2/week	8	---	Core symptom	Yes
Jang et al. (2015) (78)	Korea	Self-control	20	19/1	6–13	Hippotherapy	NR	40	2/week	12	---	Core symptom	None
Schoenfelder et al. (2017) (79)	United States	Self-control	11	6/5	14–18	Usual physical activity	NR	NR	7/week	4	---	Core symptom	None
Siu et al. (2020) (80)	Hong Kong	Self-control	14	10/4	7–9	Rugby	NR	90	1/week	6	---	Core symptom	NR
Shema-Shiratzky et al. (2019) (81)	Israel	Self-control	14	11/3	8–12	Treadmill	NA	30–60	3/week	6	---	Executive function; Core symptom	None
Smith et al. (2013) (82)	United States	Self-control	17	NR	5–9	Aerobic and motor skill	MVPA	30	7/week	8	---	Executive function; Core symptom	None
Pontifex et al. (2013) (83)	United States	Self-control	20	14/6	8–10	Running	Moderate	20	1/week	1	---	Executive function	None
Piepmeier et al. (2015) (11)	United States	Self-control	14	9/5	8–14	Cycling on an ergometer	Moderate	30	1/week	1	---	Executive function	Yes
Hung et al. (2016) (84)	Taiwan	Self-control	20	20/0	8–12	Treadmill	moderate	30	1/week	1	---	Executive function	None
Ludyga et al. (2017) (10)	Switzerland	Self-control	16	11/5	11–16	Cycling on an ergometer	Moderate	20	1/week	1	---	Executive function	Yes
Craft (1983) (85)	United States	Self-control	31	31/0	7–10	Cycling on an ergometer	MVPA	10	4/week	1	---	Executive function	None
McKune et al. (2003) (86)	Pretoria	Self-control	13	10/3	5–13	Running and jumping	Moderate	60	5/week	5	---	Core symptom	NR

RCT, randomized controlled trial; HIIT, high intensity interval training; NR, no reported; MVPA, moderate-to-vigorous physical activity.

### 3.2. Methodological quality

Using the PEDro tool, the total score ranged from 4 to 8 points, which indicated a fair (22.7%) to good (77.3%) quality (Appendix 2 S4 Table S4). As we know, it was very difficult to meet high quality criteria in exercise studies in which neither the provider or patients nor outcome assessor could be blinded, and these studies failed to meet the blinding of participants and outcome assessment guidelines. Additionally, only one study used allocation concealment. For the JBI appraisal, we assessed one study as being of poor methodological quality because of very serious risk of bias (Appendix 2 S4 Table S5).

### 3.3. Outcome-executive functions

**Figure 2:**
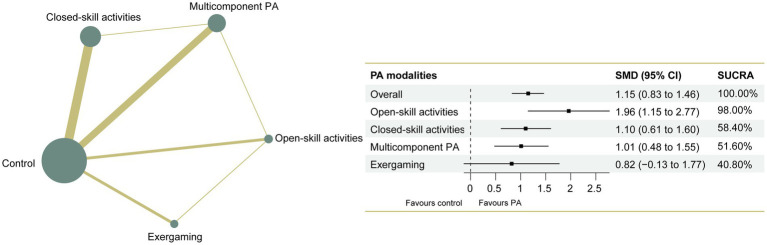
Network plot of comparisons and the efficacy of varied treatments compared with the control group for executive functions. Each node represents an intervention and its size depends on the number of participants. The connecting lines between 2 nodes represents 1 or more trials in which the 2 interventions have been compared directly. The thickness of the lines connecting 2 nodes is weighted according to the number of trials that directly compared the interventions it connected. PA, physical activity; SMD, standardized mean difference; SUCRA, the surface under the cumulative ranking curve.

**Figure 3:**
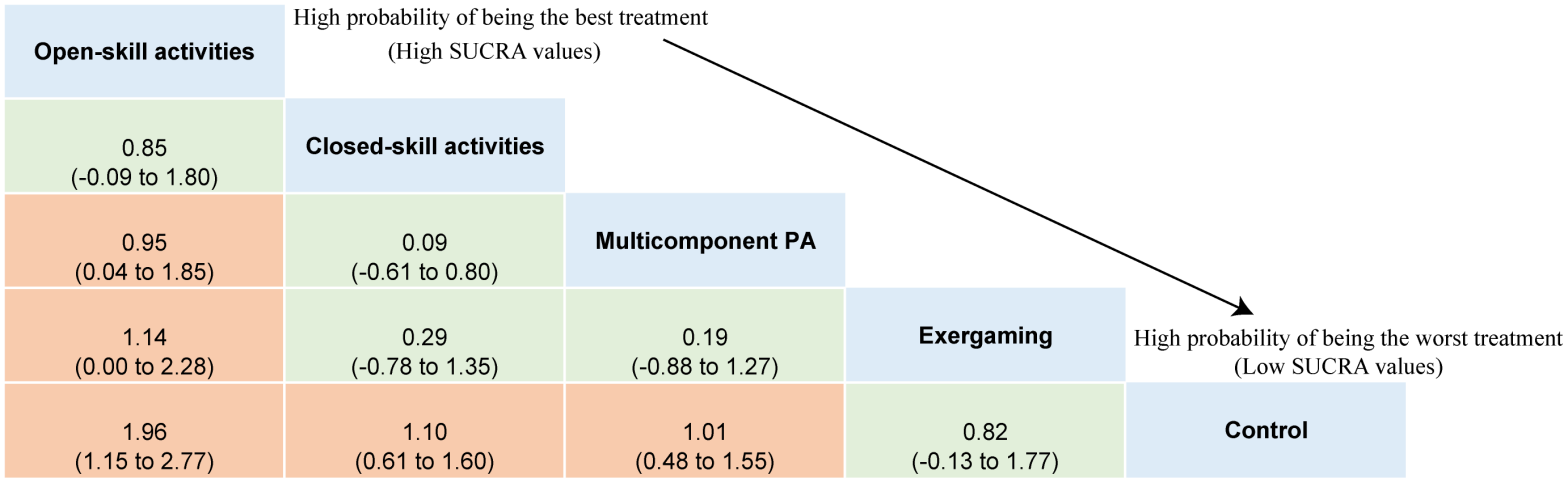
League table of outcome analyses for executive functions. PA, physical activity; SUCRA, the surface under the cumulative ranking curve.

The NMA of executive functions included 33 studies and 1,289 participants. Pairwise meta-analyses for various physical exercise and the control group comparisons were provided with detailed individual trial-level information, summarized in Appendix 2 S5 Table S6. Overall results indicated that physical exercise can effectively improve executive functions compared with the control group (SMD = 1.15, 95% CI: 0.83 to 1.46), with an overall *I*^2^ value of 79.0%. The therapeutic effects from the various physical exercise interventions ranged from medium to large. The network plot for executive functions (Figure 2) showed all the available comparisons from the included studies. In Figure 2, all the physical exercise interventions were directly compared with non-physical exercise controls, and direct comparisons between closed-skill activities and open-skill activities as well as exergaming were lacking. Inconsistency network models were used to test the global consistency of direct and indirect effects for pairwise and multi-arm comparisons simultaneously, revealing no significant global inconsistency (*p* > 0.05; Appendix 2 S6 Figure S1). The results of the network meta-analysis indicated that all types of physical exercise interventions except exergaming were superior to non-physical exercise controls, with SMDs ranging from 1.01 (95% CI: 0.48 to 1.55) for multicomponent physical exercise to 1.96 (95% CI: 1.15 to 2.77) for open-skill activities. The ranking of the physical exercise interventions based on cumulative probability plots and SUCRAs is shown in Appendix 2 S6 Figure S2. Open-skill activities showed the greatest improvement in executive functions, with a SUCRA value of 98.0% (Figure 2). The league table of outcome analyses for executive functions is given in Figure 3. Our subgroup analysis on three core components of executive functions revealed some inconsistent results (Appendix 2 S7 Figures S3–S13). For inhibitory control, open-skill activities were still the most promising physical exercise type, with a SUCRA value of 99.1% (SMD = 1.94, 95% CI: 1.24 to 2.64). Closed-skill activities possessed the greatest likelihood of being the best intervention for working memory (SUCRA = 75.9%, SMD = 1.21, and 95% CI: −0.22 to 2.65), along with the suboptimal intervention multicomponent physical exercise (SUCRA = 71.3%, SMD = 1.05, and 95% CI: 0.19 to 1.92). However, for cognitive flexibility, multicomponent physical exercise (with a mean rank of 2.2) had a slightly higher probability of being the most promising physical exercise treatment (SUCRA = 70.3%, SMD = 1.44, and 95% CI: −0.19 to 3.07). The sparseness of the network led to wide confidence intervals for some treatment comparisons. Sensitivity analyses showed that the significant result of this network meta-analysis was not unduly influenced by the study quality. Comparison-adjusted funnel plots failed to provide evidence of obvious publication bias (Appendix 2 S8 Figures S14–16). Based on the results of the meta-regression analyses, no significant modifying effects were found for the year of study, study design and quality, ages, medication use, frequency, intensity, duration, and length of physical exercise intervention, indicating that the assumption of transitivity was upheld. The meta-regression analysis results can be found in Appendix 2 S9 Figure S17. The certainty of this evidence was judged to be high to very low overall (Appendix 2 S10 Table S7).

### 3.4. Outcome-major symptoms

**Figure 4:**
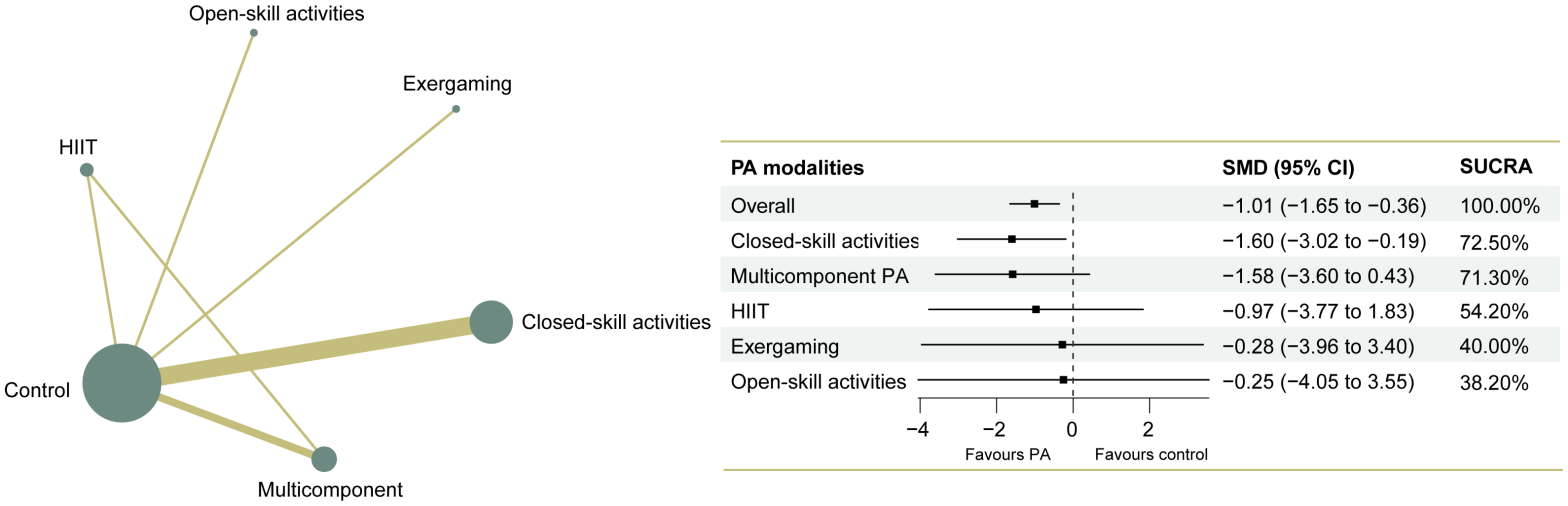
Network plot of comparisons and the efficacy of various treatments compared with the control group for hyperactivity/impulsivity. Each node represents an intervention and its size depends on the number of participants. The connecting lines between 2 nodes represents 1 or more trials in which the 2 interventions have been compared directly. The thickness of the lines connecting 2 nodes is weighted according to the number of trials that directly compared the interventions it connected. PA, physical activity; SMD, standardized mean difference; SUCRA, the surface under the cumulative ranking curve.

**Figure 5:**
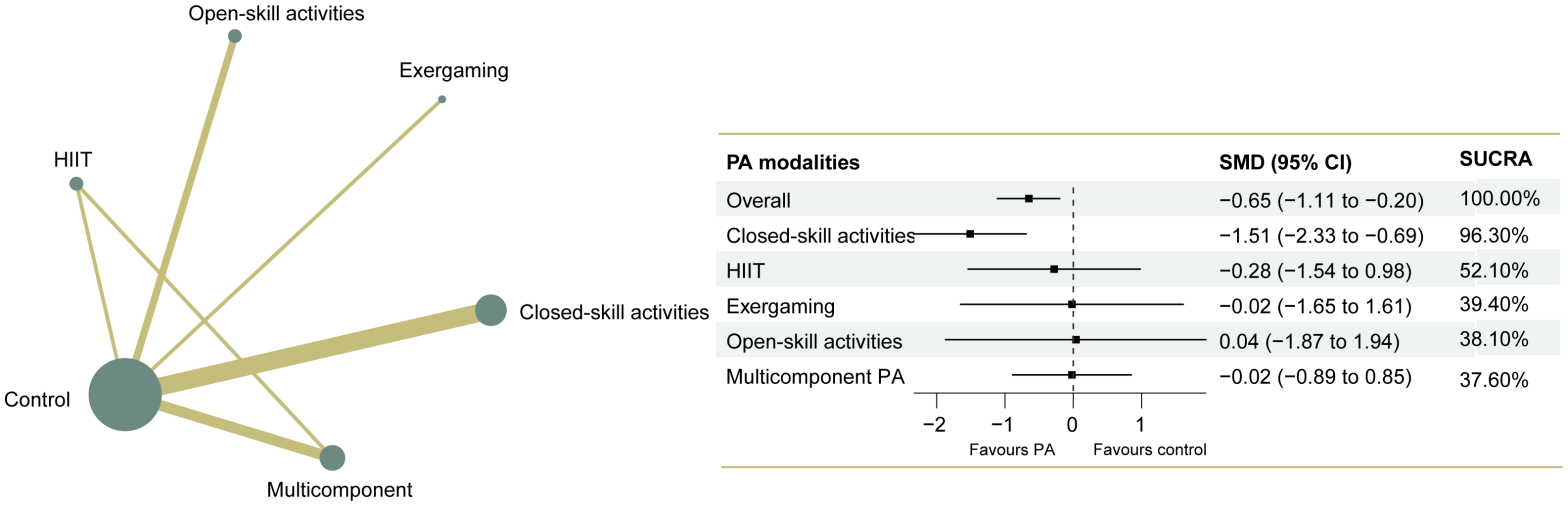
Network plot of comparisons and the efficacy of varied treatments compared with the control group for inattention. Each node represents an intervention and its size depends on the number of participants. The connecting lines between 2 nodes represents 1 or more trials in which the 2 interventions have been compared directly. The thickness of the lines connecting 2 nodes is weighted according to the number of trials that directly compared the interventions it connected. PA, physical activity; SMD, standardized mean difference; SUCRA, the surface under the cumulative ranking curve.

**Figure 6:**
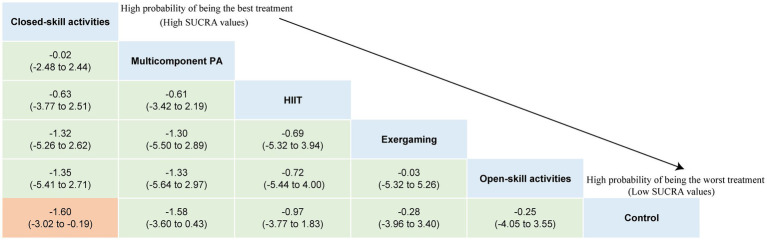
League table of outcome analyses for hyperactivity/impulsivity. PA, physical activity; SUCRA, the surface under the cumulative ranking curve.

**Figure 7:**
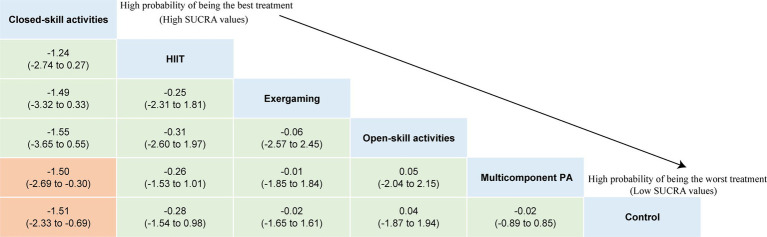
League table of outcome analyses for inattention. PA, physical activity; SUCRA, the surface under the cumulative ranking curve.

The NMA on major symptoms included 18 studies and 823 participants. Pairwise meta-analyses for various physical exercise and the control group comparisons were summarized in Appendix 2 S5 Table S6. Overall findings showed that physical exercise interventions were effective at improving hyperactivity/impulsivity (SMD = −1.01, 95% CI: −1.65 to −0.36) symptoms with a large effect, and they improve inattention (SMD = −0.65, 95% CI: −1.11 to −0.20) symptoms with a moderate effect, with overall *I*^2^ values of 88.0 and 81.0%, respectively. The network plots of the intervention comparisons are shown in Figures 4, 5, which indicated that all the physical exercise interventions were directly compared with non-physical exercise controls. No significant inconsistency was found in hyperactivity/impulsivity (Appendix 2 S11 Figure S18) and inattention (Appendix 2 S12 Figure S20), and the assumption of consistency was satisfied for the overall level of each treatment (*p* > 0.05). The results of our network meta-analysis suggested that closed-skill activities possessed the greatest likelihood of being the best intervention, with a SUCRA value of 72.5% for hyperactivity/impulsivity (SMD = −1.60, 95% CI: −3.02 to −0.19) and 96.3% for inattention (SMD = −1.51, 95% CI: −2.33 to −0.69). League tables of outcome analyses for hyperactivity/impulsivity and inattention are presented in Figures 6, 7. The ranking of the physical exercise interventions based on cumulative probability plots and SUCRAs is shown in Appendix 2 S11 Figure S19 and Appendix 2 S12 Figure S21. Sensitivity analyses showed that the results of this network meta-analysis were stable. We did not detect obvious publication bias based on the funnel plots (Appendix 2 S13 Figures S22, S23). Transitivity was explored using meta-regression sensitivity analyses. The meta-regression results indicated that frequency and duration per session were moderators on the effects of closed-skill activities for major symptoms (Appendix 2 S14 Figures S24, S25). The certainty of this evidence was judged to be high to very low overall (Appendix 2 S15 Tables S8, S9). A partially contextualized framework used for the classification of interventions based on network meta-analysis is shown in Appendix 2 S16 Table S10, showing no conflicting results were yielded.

## 4. Discussion

Attention-deficit/hyperactivity disorder is a neurobiologically based condition that interferes with a person’s ability to sustain attention, control impulsive behavior, and focus on a task (41). Seiffer et al. revealed that moderate-intensity physical activity can be an alternate treatment for ADHD (19, 24). Our previous studies also confirmed that physical exercise intervention could relieve ADHD-related symptoms and improve executive functions (16, 17). However, previously published literature lacks a comparison of different physical exercise modalities for children and adolescents with ADHD. The results from this network meta-analysis reconfirmed the beneficial effects of various physical exercise interventions on executive functions and ADHD-related symptoms and provided little evidence that open-skill activities which require participants to react in a dynamically changing and externally paced environment had the highest probability of being the most promising physical exercise treatment for improving executive functions, particularly in inhibitory control, while closed-skill activities dominated by aerobic exercises tended to be the most effective in helping working memory and multicomponent physical exercise tended to be the most effective in helping cognitive flexibility. Closed-skill activities dominated by aerobic exercises possessed the greatest likelihood of being the best intervention for ADHD-related symptoms. Our findings should be interpreted with caution, because limited quality and direct evidence were highly represented among the included studies.

According to empirical studies, participating in physical activity boosts arousal levels, cerebral blood flow, and neurotransmitter production (e.g., dopamine), all of which help to improve cognitive functions (87). Engaging in physical activity and exercise also provides a learning experience necessary for proper cognitive development, and these learning processes may inherently reinforce executive functions (88). For instance, when children are playing football, they need to pay attention, remember instructions, and often inhibit irrelevant behaviors from the environment, ensuring that they perform a particular movement or activity successfully (69), which is closely related to the use of executive functions, such as inhibitory control (17, 43). For ADHD-related symptoms, studies have reported that exercises might augment the synthesis and release of dopamine and other catecholamines in the prefrontal cortex, nucleus accumbens, caudate nucleus, and basal ganglia (89), and the underlying mechanism for physical exercise interventions to relieve inattentive symptoms may be primarily associated with increased dopamine levels in the brain.

The primary distinction in earlier studies was the type of physical activity, ranging from racket sports to aerobics and high-intensity interval training to exergames. In contrast, we conducted a network meta-analysis comparing different physical exercise modalities for children and adolescents with ADHD to provide the best evidence of efficacy. In this study, we did not categorize physical exercise modalities as aerobic, resistance and endurance training in the traditional way but innovatively divided them into open-skill and closed-skill activities. It is reasonable to assume that this is a more child-friendly classification method. It has been proposed that the environment within which the activity is performed is the primary difference between open and closed skill exercises (90). When compared to closed-skill activities, open-skill activities demand a higher level of executive control in advance anticipation (e.g., online updating of external changes and predicting related outcomes) and imperative responses (e.g., initiating in-time actions in response to external changes, including the initiation of appropriate actions and the inhibition of inappropriate actions) due to the changing environment and diversity of responses under high time pressure (91). In contrast, closed-skill activities make individuals tend to follow set patterns and hence be more consistent. Our results preliminarily indicated that open-skill activities were more beneficial for EFs, particularly in inhibitory control. Ball sports are typical open-skill activities, and it is postulated that exercise and sports associated with cognitively challenging tasks (e.g., table tennis and soccer) have a significant impact on executive functions (87). These findings were consistent with our previous findings that 6 weeks of football exercise helped to improve the executive functions of ADHD children, primarily in inhibitory control. Open-skill activities require participants to exercise in a dynamic, unpredictable, and external rhythm environment, which has higher requirements for their sports coordination ability. The higher the requirement of the sports coordination ability for the involved movement, the higher the cognitive load given to the brain, and therefore, the nervous system will be more stimulated. Therefore, long-term participation in open-skill activities may cause the neural network to be more closely connected, thus stimulating the enhancement of inhibitory function (92). Furthermore, it is worth mentioning that executive function is a generic term for a series of advanced cognitive functions, including planning, inhibition, self-regulation, flexibility and other components, which is categorized into three elements: inhibitory control, working memory, and cognitive flexibility, of which inhibitory control is the most essential component (93). Consistent with a previous meta-analysis, physical exercise interventions resulted in the largest effect size on inhibitory control (94), and most forms of physical exercise were effective at improving inhibitory control. Participants need to use their inhibitory control to follow instructions and stay on task when they participate in physical exercise, and inhibitory control plays a critical role for young children, in which it emerges first as the ability to ignore irrelevant stimuli due to the nature of environmental distractions (95, 96). When youths have reached a particular degree of inhibitory control development, they may be better able to use other executive function elements, such as working memory and shifting (95, 96). Furthermore, inhibitory control may be more sensitive than other EF components (e.g., switching) to the effect of physical exercise during childhood (97). In fact, the bulk of the included studies in the current meta-analysis were on children and adolescents, which may be one of the reasons why the largest effect on inhibitory control was found (87).

Working memory is the ability to hold things in mind (98). For the working memory of EFs, closed-skill activities showed significant effects and were superior to others based on their SUCRA values. Most of the closed-skill activities mentioned in our included studies were aerobic exercises, including simple running, treadmill exercise, and cycling on an ergometer. The improvement in working memory is related to the enhancement of aerobic fitness. Some studies have shown that working memory and the level of brain activation in young students significantly improve after acute and chronic aerobic exercise (92). In our network meta-analysis results, the first-ranked closed-skill activities were not significant, but the second-ranked multicomponent physical exercise was significant when compared to the control group. According to our current understanding, in the network meta-analysis, the ranking based on the SUCRA value and the significance of relative effects based on the point estimate and confidence interval are independent of one another. Ranking the effectiveness of interventions is a major strength of network meta-analysis, and the SUCRA method is most frequently used. Nevertheless, SUCRA does not consider the magnitude of differences in effects between treatments, particularly in a simulation in which the first-ranked treatment may be only slightly better than the second-ranked treatment (99). One contributing factor may be attributed to the complexity of the network meta-analysis, in which some single interventions may result in a significant (or nonsignificant) effect; however, this interpretation is weakened when considering other interventions in a comparative analysis, especially when the network meta-analysis involves more than three or four interventions, and the cognitive challenge of optimally interpreting these evidence summaries is daunting (99, 100). As we mentioned before, the confidence interval crossing the null would be irrelevant in the NMA, because the NMA is more likely to have wide and imprecise estimates.

For the cognitive flexibility of EFs, multicomponent physical exercise showed significant effects superior to others based on SUCRA values. Cognitive flexibility refers to the cognitive function of switching from one mode of behavior and pattern of thinking to another (101), which plays a fundamental role in almost all complex cognitive behaviors. The variety and complexity of task transformations help facilitate the cognitive processing of cognitive flexibility. Previous studies justified that mixed exercises were more likely to yield beneficial training effects on children’s EFs than aerobic exercise alone (102). Alvarez-Bueno et al. found that qualitatively enriched and quantitatively enhanced exercise benefitted different cognitive domains (103). Compared to the single form of physical exercise, multicomponent physical exercise is richer and requires a higher degree of cognitive processing. Some mechanisms have been proposed to explain the effects of multicomponent physical exercise on cognitive function, which suggests that the impact of multicomponent physical exercise on cognition implies different paths and supports the existence of a complex brain axis. The association between multicomponent physical exercise and brain-derived neurotrophic factor (BDNF), a factor that promotes the growth and differentiation of neurons and supports the survival of existing neurons, was previously explored (104). Multicomponent physical exercise is the most recommended type of physical intervention in older adults (104), and whether it is most effective in supporting cognitive flexibility in ADHD children deserves further exploration by direct evidence comparison.

Physical exercise can increase coordination between the brain regions necessary for distributing attention and other specific areas, assisting children with ADHD in processing external information and enhancing attention. Regular physical exercise can also promote the activity of adrenal hormone receptors and enhance dopamine and norepinephrine production and secretion, resulting in improved concentration. Previous research demonstrated that mixed exercises were more likely than single aerobic exercise to relieve the symptoms of ADHD (102). This result was not consistent with our findings. Our analyses revealed that closed-skill activities, which were accessible in daily life, ranked first instead of multicomponent physical exercise. It may be that regular closed-skill physical exercise helps children with ADHD form a directional action pattern, standardize their behavior, and become less impulsive and aggressive (24). In a rat model of ADHD, one study found that swimming could reduce hyperactivity, impulsivity, and aggressive behavior while also improving short-term memory. The possible mechanism by which swimming ameliorates ADHD symptoms occurs by upregulating dopamine levels and downregulating dopamine D2 receptor expression (105). There is a lack of dopamine in an ADHD brain, and exercising provides a natural dopamine boost that can ease symptoms of ADHD. Aerobic exercise is a great way to regulate dopamine levels. Additionally, there may be a number of factors that limit the effectiveness of multicomponent physical exercise. First, it might be challenging to guarantee that each exercise component is performed for the recommended duration and frequency when several are performed consecutively and sequentially, which in turn could weaken its positive effects (21). In addition, combining different exercise components could make the intervention more complicated to conduct and have a negative influence on intervention fidelity (the consistency between plan and execution) (21), especially for ADHD children. Physical activities are entertaining and well organized, which also helps with concentration, and minors may be more inclined to enjoy activities that are fun and simple. This is also the reason why no significant effect of HIIT was observed in our study. HITT is a form of exercise known as interval training comprises repeated bursts of high-intensity effort and a range of recuperation durations (13). It is hypothesized that it may make children bored and tired. The content of the physical exercise should increase the motivation and participation of children with ADHD (13).

In our study, we searched multiple databases to ensure that we identified as many relevant studies as possible. In addition, there were no publication restrictions for the included studies, and studies published in English and other languages were within the range of our analysis, thereby increasing the strength of the analysis. However, some limitations must be considered when interpreting our findings. First, the number of studies focused on ADHD-related symptoms in children and adolescents is still limited. In addition, compared with the direct comparison, the statistical power of the indirect comparison is lower, the range of the confidence interval is wider, and the more common controls were passed through indirect comparisons, in which the analysis error is also increased (106). To provide more direct evidence about the relative effectiveness of the various physical exercise therapies, more multi-arm RCTs should be conducted in the future. Second, the findings may be subject to different measurement methods, leading to heterogeneous results. Although we used subgroup analysis and meta-regression analysis to explore some of the sources of heterogeneity, there were nevertheless some moderators at the individual level. Third, the optimal dose and minimal threshold for the beneficial effect of physical exercise on executive functions and major symptoms in children and adolescents with ADHD have not been substantially discussed in the literature reviewed.

The findings in our study have some important implications for public health and education. Physical exercise is malleable, and improved physical exercise can result in improved executive functions and major symptoms, which may lead to better performance in school and home and even better quality of life for children and adolescents with ADHD (87, 107). Therefore, the need for physical exercise opportunities should be highlighted for individuals with ADHD. Furthermore, the choice and design of the physical exercise modality should be individualized based on the children’s current level of function deficits, simultaneously taking into account the fun of the physical exercise and attracting the attention of children.

## 5. Conclusion

Physical exercise can effectively improve executive functions and alleviate major symptoms in children and adolescents with ADHD with no side effects. Our preliminary findings revealed that open-skill activities requiring participants to react in a dynamically changing and externally paced environment induced the greatest benefits for executive functions, particularly in terms of inhibitory control. In contrast, closed-skill activities dominated by aerobic exercises tended to be the most effective in supporting working memory, and multicomponent physical exercise tended to be the most effective in promoting cognitive flexibility. Closed-skill activities dominated by aerobic exercises might be more advantageous for hyperactivity/impulsivity and inattention symptom improvement. Future interventions should consider these findings during the design of physical exercise interventions and programs. Nevertheless, these results should be interpreted with caution given the limitations of our meta-analysis mentioned above.

## Data availability statement

The original contributions presented in the study are included in the article/Supplementary material, further inquiries can be directed to the corresponding author.

## Author contributions

FZ and YR conceived and designed the study. FZ, XZ, XB, and DK collected the data and performed the analysis. BL, YY, and JZ assisted with the investigation. FZ, YR, and BL wrote and revised the manuscript. All authors contributed to the article and approved the submitted version.

## Funding

This research was funded by the Ministry of Education of Humanities and Social Science Foundation (21YJA890025) and Interdisciplinary Research Fundation for Doctoral Candidates of Beijing Normal University (BNUXKJC2212) and National Key Research and Development Program of China (2017YFC1311101).

## Conflict of interest

The authors declare that the research was conducted in the absence of any commercial or financial relationships that could be construed as a potential conflict of interest.

## Publisher’s note

All claims expressed in this article are solely those of the authors and do not necessarily represent those of their affiliated organizations, or those of the publisher, the editors and the reviewers. Any product that may be evaluated in this article, or claim that may be made by its manufacturer, is not guaranteed or endorsed by the publisher.

## Footnotes

^1^ www.clinicaltrials.gov
