# Supplementary material for: Comparative effectiveness of various physical exercise interventions on executive functions and related symptoms in children and adolescents with attention deficit hyperactivity disorder: A systematic review and network meta-analysis
Source: Front Public Health. 2023 Mar 24;11:1133727. doi: 10.3389/fpubh.2023.1133727 (PMC10080114; doi:10.3389/fpubh.2023.1133727)
Supplement: Supplementary file 2 [file Data_Sheet_2.pdf]

Appendix 2

Comparative effectiveness of various physical exercise interventions on executive functions and related symptoms in children and adolescents with attention deficit hyperactivity disorder: A systematic review and network meta-analysis

Feilong Zhu[1], Xiaotong Zhu[1], Xiaoyu Bi[1], Dongqing Kuang[1], Boya Liu[2], Jingyi Zhou[1], Yiming Yang[1], Yuanchun Ren[1]*

[1] *College of Physical Education and Sports, Beijing Normal University, China*

[2] *School of Social Ecology, University of California, Irvine, USA*

* Corresponding author

Yuanchun Ren

Email: yuanchun-ren@bnu.edu.cn

S1. Updated protocol

Table S1 Protocol deviations

Section	Previous protocol	Update
Title	Comparative efficacy of physical activity modalities in children and adolescents with attention deficit/hyperactivity disorder: A systematic review and network meta-analysis	Comparative effectiveness of various physical exercise interventions on executive functions and related symptoms in children and adolescents with attention deficit hyperactivity disorder: A systematic review and network meta-analysis
Authors	Feilong Zhu, Xiaotong Zhu, Yu Wu, Xiaoyu Bi, Dongqing Kuang, Yuanchun Ren	Feilong Zhu, Xiaotong Zhu, Xiaoyu Bi, Dongqing Kuang, Boya Liu, Jingyi Zhou, Yuanchun Ren
Search strategy	With no limitation on language and publication data, the databases for Web of Science, PubMed, Embase, Cochrane Central Register of Controlled Trials, SPORTDiscus, PEDro,	We searched the Web of Science, PubMed, Embase, Cochrane Central Register of Controlled Trials, SPORTDiscus, PsycINFO, CNKI and clinical trials databases without

	ScienceDirect, PsycINFO and ClinicalTrials.gov was searched.	restrictions on language.
Study quality assessment	Risk of bias was assessed by 2 authors according to the Cochrane Collaboration's risk-of-bias tool, which consists of seven items: random sequence generation, allocation concealment, blinding of participants and personnel, blinding of outcome assessors, incomplete outcome data, incomplete outcome reporting, and other bias.	The methodological quality of included randomized controlled trials and non-randomized controlled studies was assessed by 2 reviewers using the Physiotherapy Evidence Database scale (PEDro).
Data synthesis	No meta regression analysis.	We also conducted meta regression analysis (with frequency, intensity, duration, and length of PA interventions as covariates) for the primary outcomes.

S2. Search strategy

PubMed 549

#1 "Attention Deficit Disorder with Hyperactivity"[Mesh] OR Attention Deficit Disorders with Hyperactivity[tiab] OR Attention Deficit Hyperactivity Disorder*[tiab] OR Hyperkinetic Syndrome*[tiab] OR ADDH[tiab] OR ADHD[tiab] OR Attention Deficit Disorder*[tiab] OR ADD[tiab] OR Hyperactivit*[tiab] OR Inattenti*[tiab] OR Impulsivit*[tiab] OR attention-deficit hyperactivity disorder*[tiab] OR Attention Deficit-Hyperactivity Disorder*[tiab] OR Hyperkinet*[tiab] OR Overactive*[tiab]

#2 "Exercise"[Mesh] OR "Sports"[Mesh] OR Exercise*[tiab] OR Physical Activit*[tiab] OR Physical Exercise*[tiab] OR Isometric Exercise*[tiab] OR Aerobic Exercise*[tiab] OR Exercise Training*[tiab] OR Sport*[tiab] OR Movement[tiab] OR Workout*[tiab] OR Physical Training*[tiab] OR Energy expenditure[tiab] OR Athletic*[tiab] OR Acute Exercise*[tiab] OR Chronic Exercise*[tiab] OR Exercise intervention[tiab] OR Intervention* [tiab] OR Training*[tiab] OR Program[tiab] OR Physical education[tiab] OR Locomotor Activit*[tiab] OR Physical therap*[tiab] OR motor activit*[tiab] OR Soccer[tiab] OR Swim*[tiab] OR Aquatic*[tiab] OR Div*[tiab] OR Football[tiab] OR Pin pang[tiab] OR Ping-pong[tiab] OR Ping pang[tiab] OR Basketball[tiab] OR Cricket[tiab] OR Tennis[tiab] OR Rugby[tiab] OR Danc*[tiab] OR Martial art*[tiab] OR Netball[tiab] OR Hockey[tiab] OR Gym*[tiab] OR Horse rid*[tiab] OR Horseback rid*[tiab] OR Equestrian[tiab] OR Baseball[tiab] OR Yoga[tiab] OR Badminton[tiab] OR Taekwondo[tiab] OR Judo[tiab] OR Cycling[tiab] Physical fitness[tiab] OR Fitness[tiab] OR Resistance exercise[tiab] OR Bicycling[tiab] OR Boxing[tiab] OR Golf[tiab] OR Tai Ji[tiab] OR

Mountaineering[tiab] OR Racquet sports[tiab] OR Skating[tiab] OR Snow sports skiing[tiab] OR Wrestling[tiab] OR Weight lifting[tiab] OR Games recreational[tiab] OR Weight bearing exercise[tiab] OR Strength training[tiab] OR Running[tiab] OR Agility[tiab] OR Trampoline[tiab] OR Snowshoeing[tiab] OR Skating[tiab] OR Exergaming[tiab] OR Skateboarding[tiab] OR Walking[tiab] OR Treadmill[tiab]

#3 "Child"[Mesh] OR "Adolescent"[Mesh] OR "Young Adult"[Mesh] OR "Minor"[Mesh] OR Adolescen*[tiab] OR Teen*[tiab] OR Teenager*[tiab] OR Youth*[tiab] OR "Minors"[tiab] OR Minor*[tiab] OR "Child"[tiab] OR Child*[tiab] OR Kid[tiab] OR Kids[tiab] OR Girl*[tiab] OR Boy*[tiab] OR Under age*[tiab] OR Underage*[tiab] OR Young people[tiab] OR young person[tiab] OR Prepubescen*[tiab] OR Pubescen*[tiab] OR Young Adult*[tiab] OR School age[tiab] OR Preschool*[tiab] OR Student*[tiab]

#4 #1 AND #2 AND #3

Web of Science 6236

#1 TS = (“Attention Deficit Disorder with Hyperactivit*” OR “attention-deficit hyperactivity disorder*” OR “attention deficit disorder*” OR “ADHD” OR adhd OR addh OR add OR “Attention Deficit Disorder* with Hyperactivity” OR “Attention Deficit Hyperactivity Disorder*” OR “Attention Deficit-Hyperactivity Disorder*” OR “attention deficit disorder hyperactivity” OR “attention deficit” OR “child attention deficit disorder” OR hyperactiv* OR “Hyperkinetic Syndrome*” OR “syndrome* hyperkinetic” OR “hyperkinetic syndrome childhood” OR “attention deficit hyperkinetic disorder” OR “hyperkinetic disorder” OR hyperkinet* OR overactive* OR “overactive child syndrome” OR

inattenti* OR “attention problem*” OR “syndrome hyperkinetic” OR “hyperkinetic syndrome” OR “hyperactivity disorder” OR “hyperactive child syndrome” OR “childhood hyperkinetic syndrome”)

#2 TS = (Attention Deficit Disorder with Hyperactivity)

#3 TS = (Sport* OR Exercis* OR “Locomotor Activit*” OR “Physical* therap*” OR “physical* activit*” OR “motor activit*” OR Soccer OR Swim* OR Aquatic* OR Dive OR Diving OR Football OR Pin pang OR Ping-pong OR Ping pang OR Basketball OR Cricket OR Tennis OR Rugby OR Danc* OR Athletic* OR “Martial art*” OR Netball OR Hockey OR Gym* OR “horse rid*” OR “horseback rid*” OR Equestrian OR Baseball OR Yoga OR Badminton OR Taekwondo OR Danc* OR Judo OR Cycling)

#4 TS = (Sports OR Exercise)

#5 TS=(Child OR Adolescent OR Young Adult OR Minor OR Adolescen* OR Teen* OR Teenager* OR Youth* OR Minors OR Minor* OR Child* OR Kid OR Kids OR Girl* OR Boy* OR Under age* OR Underage* OR Young people OR young person OR Prepubescen* OR Pubescen* OR Young Adult* OR School age OR Preschool* OR Student*)

#6 TS = (Child OR Adolescent OR Young Adult OR Minor)

#7 #1 OR #2

#8 #3 OR #4

#9 #5 OR #6

#10 #7 AND #8 AND#9

Cochrane Library 1164

#1 (Attention Deficit Disorder with Hyperactivit* OR attention-deficit hyperactivity disorder* OR attention deficit disorder* OR ADHD OR adhd OR addh OR add OR Attention Deficit Disorder* with Hyperactivity OR Attention Deficit Hyperactivity Disorder* OR Attention Deficit-Hyperactivity Disorder* OR attention deficit disorder hyperactivity OR attention deficit" OR child attention deficit disorder OR hyperactiv* OR Hyperkinetic Syndrome* OR syndrome* hyperkinetic OR hyperkinetic syndrome childhood OR attention deficit hyperkinetic disorder OR hyperkinetic disorder OR hyperkinet* OR overactive* OR "overactive child syndrome OR inattenti* OR attention problem* OR syndrome hyperkinetic OR hyperkinetic syndrome OR hyperactivity disorder OR hyperactive child syndrome OR childhood hyperkinetic syndrome): ti, ab, kw

#2 Attention Deficit Disorder with Hyperactivity [Mesh]

#3 (Sport* OR Exercis* OR "Locomotor Activit*" OR "Physical* therap*" OR "physical* activit*" OR "motor activit*" OR Soccer OR Swim* OR Aquatic* OR Dive OR Diving OR Football OR Pin pang OR Ping-pong OR Ping pang OR Basketball OR Cricket OR Tennis OR Rugby OR Danc* OR Athletic* OR "Martial art*" OR Netball OR Hockey OR Gym* OR "horse rid*" OR "horseback rid*" OR Equestrian OR Baseball OR Yoga OR Badminton OR Taekwondo OR Danc* OR Judo OR Cycling): ti, ab, kw

#4 Sports [Mesh]

#5 Exercise [Mesh]

#6 (Child OR Adolescent OR Young Adult OR Minor OR Adolescen* OR Teen* OR Teenager* OR Youth* OR Minors OR Minor* OR Child* OR Kid OR Kids OR Girl* OR Boy* OR Under age* OR Underage* OR Young people OR young

person OR Prepubescen* OR Pubescen* OR Young Adult* OR School age OR Preschool* OR Student*): ti, ab, kw

#7 Child [Mesh] OR Adolescent [Mesh] OR Young Adult [Mesh] OR Minor [Mesh]

#8 #1 OR #2

#9 #3 OR #4 OR #5

#10 #6 OR #7

#11 #8 AND #9 AND #10

Embase 2653

#1 'attention deficit disorder with hyperactivit*' OR 'attention-deficit hyperactivity disorder*' OR 'attention deficit disorder*' OR 'adhd' OR adhd OR addh OR add OR 'attention deficit disorder* with hyperactivity' OR 'attention deficit hyperactivity disorder*' OR 'attention deficit-hyperactivity disorder*' OR 'attention deficit disorder hyperactivity' OR 'attention deficit' OR 'child attention deficit disorder' OR hyperactiv* OR 'hyperkinetic syndrome*' OR 'syndrome* hyperkinetic' OR 'hyperkinetic syndrome childhood' OR 'attention deficit hyperkinetic disorder' OR 'hyperkinetic disorder' OR hyperkinet* OR overactive* OR 'overactive child syndrome' OR inattenti* OR 'attention problem*' OR 'syndrome hyperkinetic' OR 'hyperkinetic syndrome' OR 'hyperactivity disorder' OR 'hyperactive child syndrome' OR 'childhood hyperkinetic syndrome'

#2 'attention deficit disorder'/exp

#3 sport* OR exercis* OR 'locomotor activit*' OR 'physical* therap*' OR 'physical* activit*' OR 'motor activit*' OR soccer OR swim* OR aquatic* OR dive OR

diving OR football OR ‘pin pang’ OR ‘ping pong’ OR ‘ping pang’ OR basketball OR cricket OR tennis OR rugby OR athletic* OR ‘martial art*’ OR netball OR hockey OR gym* OR ‘horse rid*’ OR ‘horseback rid*’ OR equestrian OR baseball OR yoga OR badminton OR taekwondo OR danc* OR judo OR cycling

#4 ‘sport’/exp

#5 ‘exercise’/exp

#6 Child OR Adolescent OR Young Adult OR Minor OR Adolescen* OR Teen* OR Teenager* OR Youth* OR Minors OR Minor* OR Child* OR Kid OR Kids OR Girl* OR Boy* OR Under age* OR Underage* OR Young people OR young person OR Prepubescen* OR Pubescen* OR Young Adult* OR School age OR Preschool* OR Student*

#7 ‘Child’/exp

#8 ‘Adolescent’/exp

#9 #1 OR #2

#10 #3 OR #4 OR #5

#11 #6 OR #7 OR #8

#12 #9 AND #10 AND #11

SPORTDiscus 1765

#1 DE “Attention Deficit Disorder with Hyperactivity”

#2 (“Attention Deficit Disorder with Hyperactivit*” OR “attention-deficit hyperactivity disorder*” OR “attention deficit disorder*” OR “ADHD” OR adhd OR addh OR add OR “Attention Deficit Disorder* with Hyperactivity” OR

“Attention Deficit Hyperactivity Disorder*” OR “Attention Deficit-Hyperactivity Disorder*” OR “attention deficit disorder hyperactivity” OR “attention deficit” OR “child attention deficit disorder” OR hyperactiv* OR “Hyperkinetic Syndrome*” OR “syndrome* hyperkinetic” OR “hyperkinetic syndrome childhood” OR “attention deficit hyperkinetic disorder” OR “hyperkinetic disorder” OR hyperkinet* OR overactive* OR “overactive child syndrome” OR inattenti* OR “attention problem*” OR “syndrome hyperkinetic” OR “hyperkinetic syndrome” OR “hyperactivity disorder” OR “hyperactive child syndrome” OR “childhood hyperkinetic syndrome”). TX

#3 DE “Sports”

#4 DE “Exercise”

#5 (Sport* OR Exercis* OR “Locomotor Activit*” OR “Physical* therap*” OR “physical* activit*” OR “motor activit*” OR Soccer OR Swim* OR Aquatic* OR Dive OR Diving OR Football OR Pin pang OR Ping-pong OR Ping pang OR Basketball OR Cricket OR Tennis OR Rugby OR Danc* OR Athletic* OR “Martial art*” OR Netball OR Hockey OR Gym* OR “horse rid*” OR “horseback rid*” OR Equestrian OR Baseball OR Yoga OR Badminton OR Taekwondo OR Danc* OR Judo OR Cycling). TX

#6 DE “Child”

#7 DE “Adolescent”

#8 (Child OR Adolescent OR Young Adult OR Minor OR Adolescen* OR Teen* OR Teenager* OR Youth* OR Minors OR Minor* OR Child* OR Kid OR Kids OR Girl* OR Boy* OR Under age* OR Underage* OR Young people OR young person OR Prepubescen* OR Pubescen* OR Young Adult* OR School age OR Preschool* OR Student*). TX

#9 #1 OR #2

#10 #3 OR #4 OR #5

#11 #6 OR #7 OR #8

#12 #9 AND #10 AND #11

PsycINFO 3042

#1 "Attention Deficit Disorder with Hyperactivity" OR Attention Deficit Disorders with Hyperactivity OR Attention Deficit Hyperactivity Disorder* OR Hyperkinetic Syndrome* OR ADDH OR ADHD OR Attention Deficit Disorder* OR ADD OR Hyperactivit* OR Inattenti* OR Impulsivit* OR attention-deficit hyperactivity disorder* OR Attention Deficit-Hyperactivity Disorder* OR Hyperkinet* OR Overactive*

#2 Exercise OR Sports OR Exercise* OR Physical Activit* OR Physical Exercise* OR Isometric Exercise* OR Aerobic Exercise* OR Exercise Training* OR Sport* OR Movement OR Workout* OR Physical Training* OR Energy expenditure OR Athletic* OR Acute Exercise* OR Chronic Exercise* OR Exercise intervention OR Intervention* OR Training* OR Program OR Physical education OR Locomotor Activit* OR Physical therap* OR motor activit* OR Soccer OR Swim* OR Aquatic* OR Div* OR Football OR Pin pang OR Ping-pong OR Ping pang OR Basketball OR Cricket OR Tennis OR Rugby OR Danc* OR Martial art* OR Netball OR Hockey OR Gym* OR Horse rid* OR Horseback rid* OR Equestrian OR Baseball OR Yoga OR Badminton OR Taekwondo OR Judo OR Cycling Physical fitness OR Fitness OR Resistance exercise OR Bicycling OR Boxing OR Golf OR Tai Ji OR Mountaineering OR Racquet sports OR Skating OR Snow sports skiing OR Wrestling OR Weight lifting OR Games recreational

OR Weight bearing exercise OR Strength training OR Running OR Agility OR Trampoline OR Snowshoeing OR Skating OR Exergaming OR Skateboarding OR Walking OR Treadmill

#3 Child OR Adolescent OR "Young Adult" OR Minor OR Adolescen* OR Teen* OR Teenager* OR Youth* OR "Minors" OR Minor* OR "Child" OR Child* OR Kid OR Kids OR Girl* OR Boy* OR Under age* OR Underage* OR Young people OR young person OR Prepubescen* OR Pubescen* OR Young Adult* OR School age OR Preschool* OR Student*

#4 #1 AND #2 AND #3

S3. The inter-rater agreement for selecting studies

Table S2 The inter-rater agreement for reading the title and abstract

Reviewer 1	Reviewer 2			Total
	Exclude	Include	Unclear	
Exclude	13514	8	11	13533
Include	9	78	8	95
Unclear	14	6	10	30
Total	13537	92	29	13658

Kappa score: 0.77 (0.72-0.83)

Table S3 The inter-rater agreement for full-text review

Reviewer 1	Reviewer 2			Total
	Exclude	Include	Unclear	
Exclude	67	1	3	71
Include	1	54	0	55
Unclear	2	2	0	4
Total	70	57	3	130

Kappa score: 0.87 (0.79-0.95)

S4. Study quality assessment

Table S4 Quality assessment for RCTs and quasi-RCTs

Reference	Item 1	Item 2	Item 3	Item 4	Item 5	Item 6	Item 7	Item 8	Item 9	Item 10	Item 11	Score
Messler et al. (2018)	1	1	0	1	0	0	0	1	1	1	1	6/10
Benzing et al. (2019)	1	1	0	1	0	0	0	1	1	1	1	6/10
Bustamante et al. (2016)	1	1	0	1	0	0	0	1	1	1	1	6/10
Chang et al. (2014)	1	0	0	1	0	0	0	1	1	1	1	5/10
Choi et al. (2015)	1	1	0	1	0	0	0	1	1	1	1	6/10
Chou et al. (2017)	1	0	0	1	0	0	0	1	1	1	1	5/10
Faramarzi et al. (2016)	1	1	0	1	0	0	0	1	1	1	1	6/10
Gelade et al. (2017)	1	1	0	1	1	0	1	1	1	1	1	8/10
Kadri et al. (2019)	1	1	0	1	0	0	0	1	1	1	1	6/10
Lee et al. (2017)	1	1	0	1	0	0	0	1	1	1	1	6/10
Memarmoghaddam et al. (2016)	1	1	0	1	0	0	0	1	1	1	1	6/10
Pan et al. (2016)	1	1	0	1	0	0	0	1	1	1	1	6/10
Pan et al. (2019)	1	0	0	1	0	0	0	1	1	1	1	5/10
Rezaei et al. (2018)	1	1	0	1	0	0	0	1	1	1	1	6/10
Silva et al. (2020)	1	1	0	1	0	0	0	0	1	1	1	5/10
Smith et al. (2020)	1	1	0	1	0	0	0	1	1	1	1	6/10
Verret et al. (2012)	1	0	0	1	0	0	0	1	1	1	1	5/10
Ziereis et al. (2015)	1	1	0	1	0	0	0	1	1	1	1	6/10
Benzing et al. (2018)	1	1	0	1	1	0	0	1	1	1	1	7/10
Chang et al. (2012)	1	1	0	1	0	0	0	1	1	1	1	6/10

Reference	Item 1	Item 2	Item 3	Item 4	Item 5	Item 6	Item 7	Item 8	Item 9	Item 10	Item 11	Score
Gawrilow et al. (2013)	1	1	0	1	0	0	0	1	1	1	1	6/10
Kang et al. (2011)	1	1	0	1	0	0	0	1	1	1	1	6/10
Chang et al. (2022)	1	1	0	1	0	0	0	1	1	1	1	6/10
Smith et al. (2019)	1	1	0	1	0	0	0	1	1	1	1	6/10
Ahmed et al. (2011)	1	1	0	1	0	0	0	1	1	1	1	6/10
Hattabi et al. (2019)	1	1	0	1	0	0	0	1	1	1	1	6/10
Hoza et al. (2014)	1	1	0	1	1	0	0	1	1	1	1	7/10
Jensen et al. (2004)	1	1	0	1	0	0	0	1	1	1	1	6/10
Garcia-Gomez et al. (2016)	1	0	0	1	0	0	0	1	1	1	1	5/10
Porter et al. (1984)	1	1	0	1	0	0	0	0	1	1	1	5/10
Oh et al. (2018)	1	1	0	1	0	0	0	1	1	1	1	6/10
Liu et al. (2018)	1	1	0	1	0	0	0	1	1	1	1	6/10
Bahram et al. (2014)	1	1	0	1	0	0	0	1	1	1	1	6/10
Hattabi et al. (2021)	1	0	0	0	0	0	0	1	1	1	1	4/10
Felmet et al. (1998)	1	1	0	1	0	0	0	1	1	1	1	5/10
Gelade et al. (2018)	1	1	1	1	0	0	0	1	1	1	1	7/10
Soori et al. (2020)	1	1	0	1	0	0	0	1	1	1	1	6/10
Song et al. (2022)	1	1	0	1	0	0	0	1	1	1	1	6/10
Chen et al. (2022)	1	1	0	1	0	0	0	1	1	1	1	6/10
Xu et al. (2021)	1	1	0	1	0	0	0	1	1	1	1	6/10
Chuang et al. (2015)	1	1	0	1	0	0	0	1	1	1	1	6/10
Ludyga et al. (2020)	1	1	0	1	0	0	0	1	1	1	1	6/10

Reference	Item 1	Item 2	Item 3	Item 4	Item 5	Item 6	Item 7	Item 8	Item 9	Item 10	Item 11	Score
Silva et al. (2015)	1	0	0	1	0	0	0	1	1	1	1	5/10
Liang et al. (2022)	1	1	0	1	0	0	0	1	1	1	1	6/10

Item1, eligibility criteria; Item 2, random allocation; Item 3, concealed allocation; Item 4, baseline comparability; Item 5, blind subjects; Item 6, blind therapists; Item 7, blind assessors; Item 8, adequate follow-up; Item 9, intention-to-treat analysis; Item 10, between-group comparisons; Item 11, estimates and variability.

Table S5 Quality assessment for self-control trials

Reference	Item 1	Item 2	Item 3	Item 4	Item 5	Item 6	Item 7	Item 8	Item 9
So et al. (2017)	Yes	Unclear	Yes	Yes	Yes	Yes	Yes	Yes	Yes
Hernandez-Reif et al. (2001)	Yes	NA	NA	No	Yes	Yes	Yes	Yes	Unclear
Lufi et al. (2011)	Yes	Yes	Yes	Yes	Yes	Yes	Yes	Yes	Yes
Cuypers et al. (2011)	Yes	NA	NA	No	Yes	Yes	Yes	Yes	Yes
Jang et al. (2015)	Yes	NA	NA	No	Yes	Yes	Yes	Yes	Yes
Schoenfelder et al. (2017)	Yes	NA	NA	No	Yes	Yes	Yes	Yes	Yes
Siu et al. (2020)	Yes	NA	NA	No	Yes	Yes	Yes	Yes	Yes
Shema-Shiratzky et al. (2019)	Yes	NA	NA	No	Yes	Yes	Yes	Yes	Yes
Smith et al. (2013)	Yes	NA	NA	No	Yes	Yes	Yes	Yes	Yes
Pontifex et al. (2013)	Yes	No	NA	Yes	Yes	Yes	Yes	Yes	Yes
Piepmeier et al. (2015)	Yes	Yes	NA	Yes	Yes	Yes	Yes	Yes	Yes
Hung et al. (2016)	Yes	No	NA	Yes	Yes	Yes	Yes	Yes	Yes
Ludyga et al. (2017)	Yes	NA	NA	Yes	Yes	Yes	Yes	Yes	Yes
Craft et al. (1983)	Yes	NA	NA	No	No	Yes	Yes	Unclear	Unclear
McKune et al. (2003)	Yes	Unclear	Yes	Yes	No	Yes	Yes	Yes	Yes

Item 1, Were the causal relationships in the study clearly stated?

Item 2, Were the baselines comparable between groups?

Item 3, Were the groups receiving the same intervention besides to be validated?

Item 4, Was a control group set up?

Item 5, Were the outcome measures diversified before and after the intervention?

Item 6, Was follow-up complete and, if not, was loss to follow-up reported and addressed?

Item 7, Were the outcome measures used in the same way in each group?

Item 8, Were the outcomes measured in a valid and reliable way?

Item 9, Was appropriate statistical analysis used?

S5. Summary table of pairwise comparison results

Table S6 Direct comparison treatment effect with control group

Outcomes	Types	Trials	Participants	Effect size	I^2
Executive functions	Open-skill activities	4	110	1.94 [1.11, 2.77]	66.0
	Closed-skill activities	13	400	1.09 [0.63, 1.55]	77.0
	Multicomponent PA	10	355	0.74 [0.32, 1.17]	62.0
	Exergaming	3	129	0.56 [0.21, 0.92]	0
Hyperactivity/ impulsivity	Closed-skill activities	7	288	-1.84 [-2.67, -1.02]	81.0
	Multicomponent PA	2	115	0.16 [-0.21, 0.52]	0
	High-intensity interval training	1	43	-0.43 [-1.05, 0.19]	NA
	Exergaming	1	51	-0.28 [-0.83, 0.28]	NA
	Open-skill activities	1	14	-0.25 [-1.35, 0.85]	NA
Inattention	Closed-skill activities	5	184	-1.57 [-2.58, -0.57]	80.0
	High-intensity interval training	1	43	-0.43 [-1.05, 0.19]	NA
	Exergaming	1	51	-0.02 [-0.57, 0.53]	NA
	Multicomponent PA	3	209	0.03 [-0.24, 0.30]	0
	Open-skill activities	1	14	-0.24 [-1.33, 0.86]	NA

S6. Analysis for executive functions

Figure S1 Loop-specific heterogeneity for executive functions

Loop	IF	seIF	z_value	p_value	CI_95	Loop_Heterog_tau2
A-D-E	0.580	6.340	0.092	0.927	(0.00,13.01)	2.223
A-B-E	0.344	1.158	0.297	0.766	(0.00,2.61)	0.637
A-B-C	0.145	1.142	0.127	0.899	(0.00,2.38)	0.609

Inconsistency plot for the executive network, assuming loop-specific heterogeneity estimates.

Figure S2 The ranking of effects for executive functions

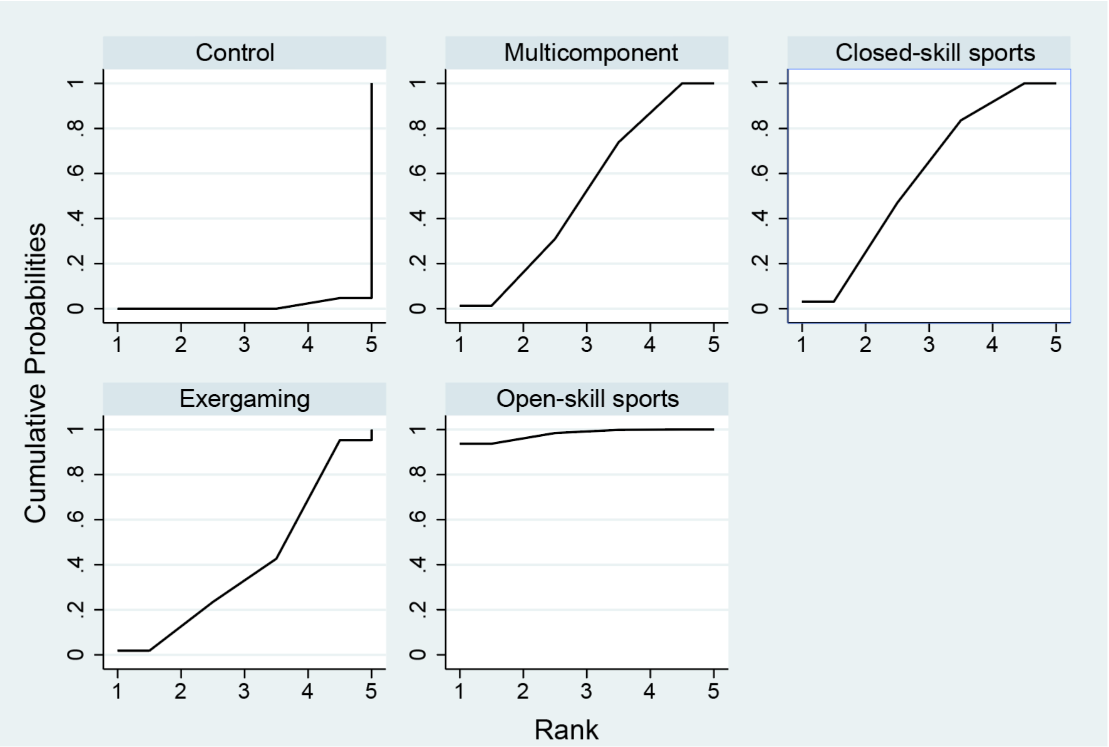

Plots of the surface under the cumulative ranking curves (SUCRA) for all comparisons in the executive functions network.

S7. Subgroup analysis for executive functions

Figure S3 Network of eligible comparisons for inhibitory control

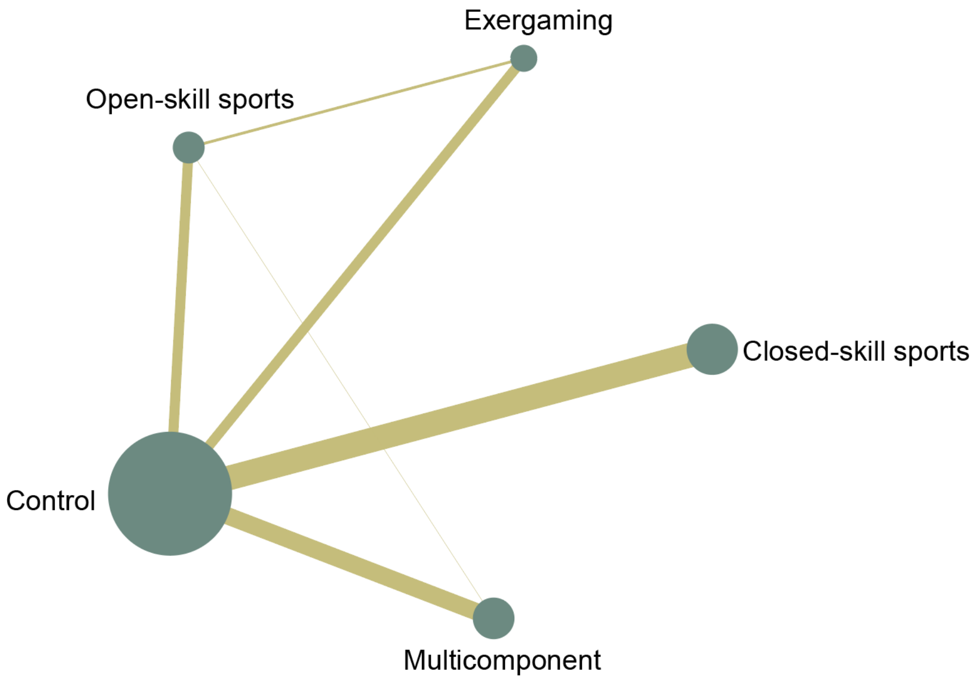

Figure S4 The ranking of effects for inhibitory control

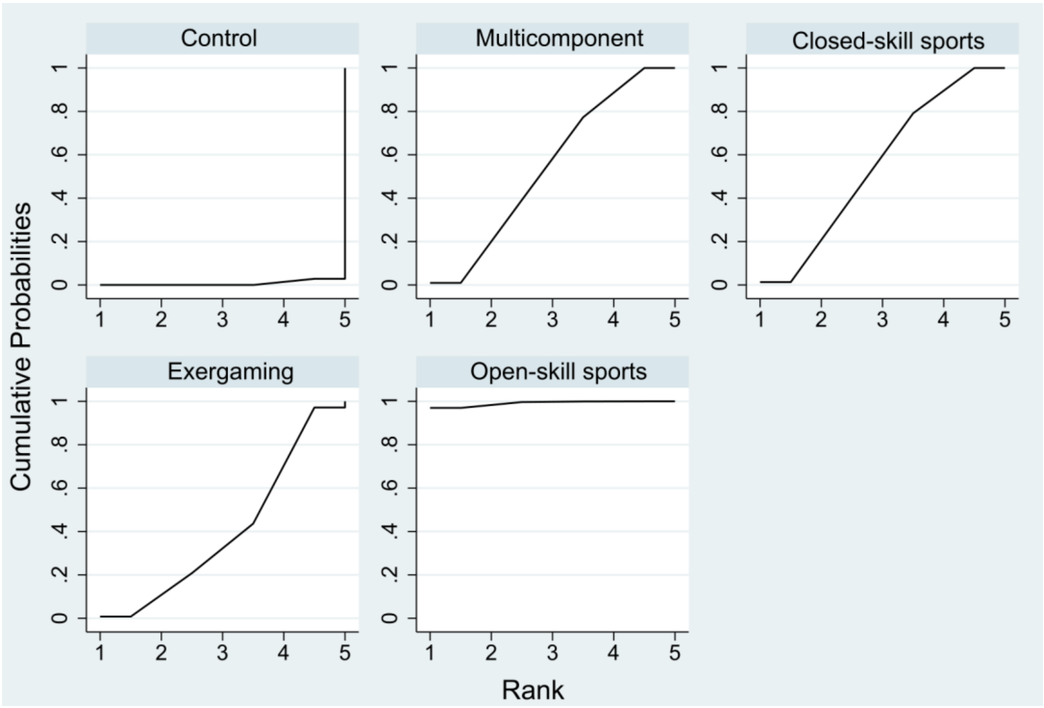

Plots of the surface under the cumulative ranking curves (SUCRA) for all comparisons in the inhibitory control.

Figure S5 Relative effect sizes of treatments efficacy based on network meta-analysis for inhibitory control

High probability of being the best treatment (High SUCRA values)

**Open-skill activities**				
0.92 (0.07 to 1.77)	**Closed-skill activities**			
0.93 (0.13 to 1.74)	0.02 (-0.71 to 0.74)	**Multicomponent PA**		
1.15 (0.18 to 2.11)	0.23 (-0.70 to 1.15)	0.21 (-0.74 to 1.16)	**Exergaming**	
1.94 (1.24 to 2.64)	1.02 (0.54 to 1.50)	1.00 (0.46 to 1.55)	0.79 (0.00 to 1.59)	**Control**

High probability of being the worst treatment (Low SUCRA values)

Figure S6 Loop-specific heterogeneity for inhibitory control

Loop	IF	seIF	z_value	p_value	CI_95	Loop_Heterog_tau2
A-D-E	0.580	6.340	0.092	0.927	(0.00,13.01)	2.223
A-B-E	0.398	1.013	0.393	0.694	(0.00,2.38)	0.512

Figure S7 Network of eligible comparisons for working memory

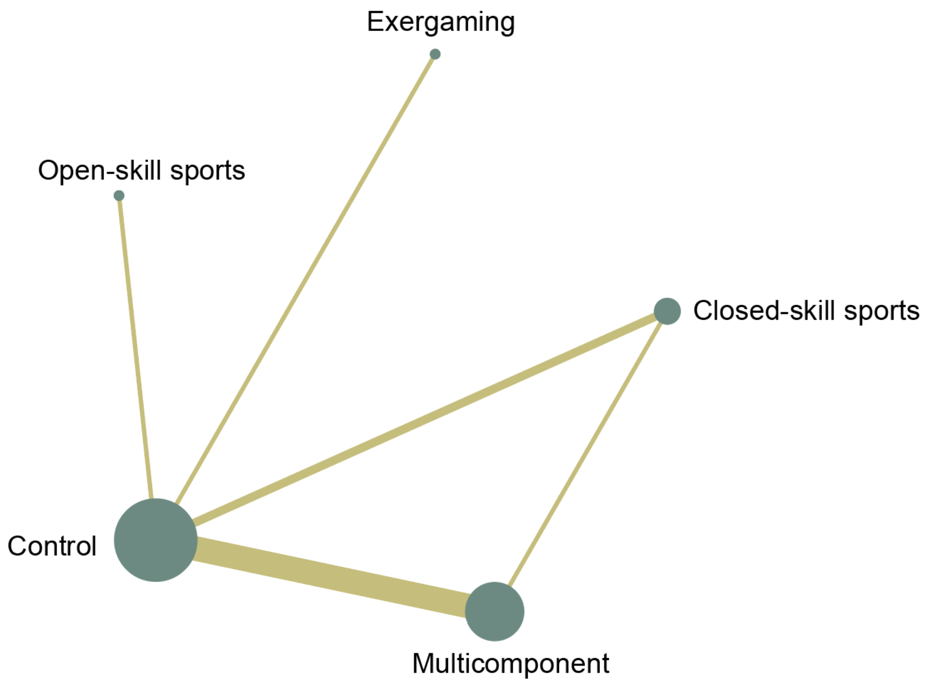

Figure S8 The ranking of effects for working memory

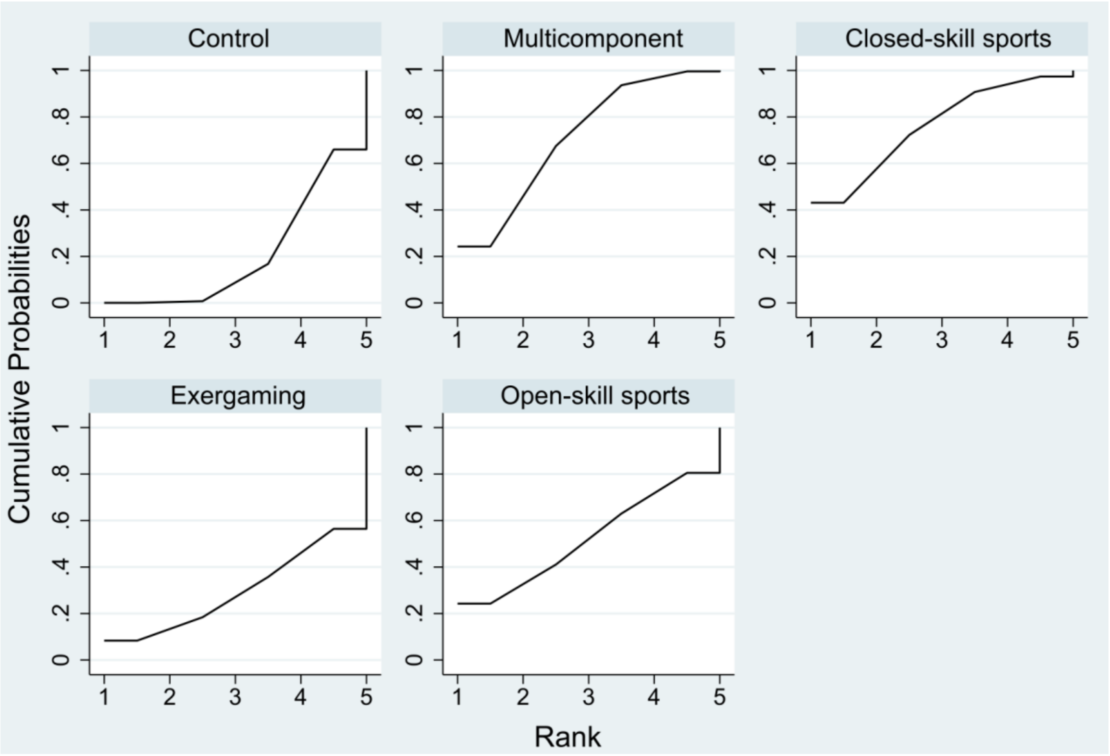

Plots of the surface under the cumulative ranking curves (SUCRA) for all comparisons in the working memory.

Figure S9 Relative effect sizes of treatments efficacy based on network meta-analysis for working memory

High probability of being the best treatment (High SUCRA values)

**Closed-skill activities**				
0.16 (-1.38 to 1.70)	**Multicomponent PA**			
0.57 (-2.09 to 3.23)	0.40 (-2.00 to 2.81)	**Open-skill activities**		
1.21 (-1.31 to 3.74)	1.05 (-1.20 to 3.30)	0.65 (-2.41 to 3.70)	**Exergaming**	
1.21 (-0.22 to 2.65)	1.05 (0.19 to 1.92)	0.65 (-1.59 to 2.89)	0.00 (-2.08 to 2.08)	**Control**

High probability of being the worst treatment (Low SUCRA values)

Figure S10 Loop-specific heterogeneity for working memory

Loop	IF	seIF	z_value	p_value	CI_95	Loop_Heterog_tau2
A-B-C	0.129	1.579	0.082	0.935	(0.00,3.22)	0.954

Figure S11 Network of eligible comparisons for cognitive flexibility

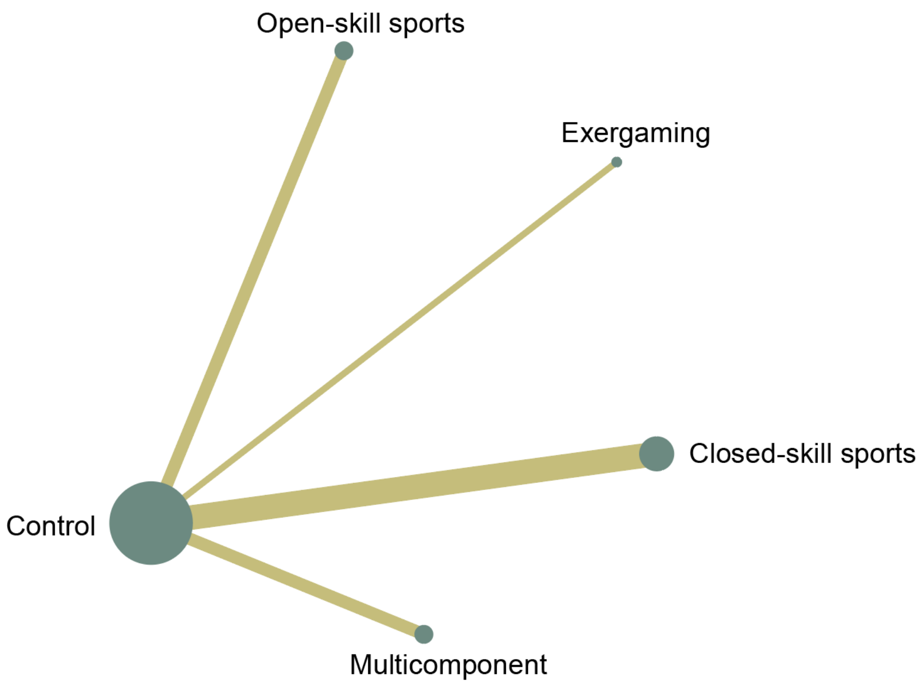

Figure S12 The ranking of effects for cognitive flexibility

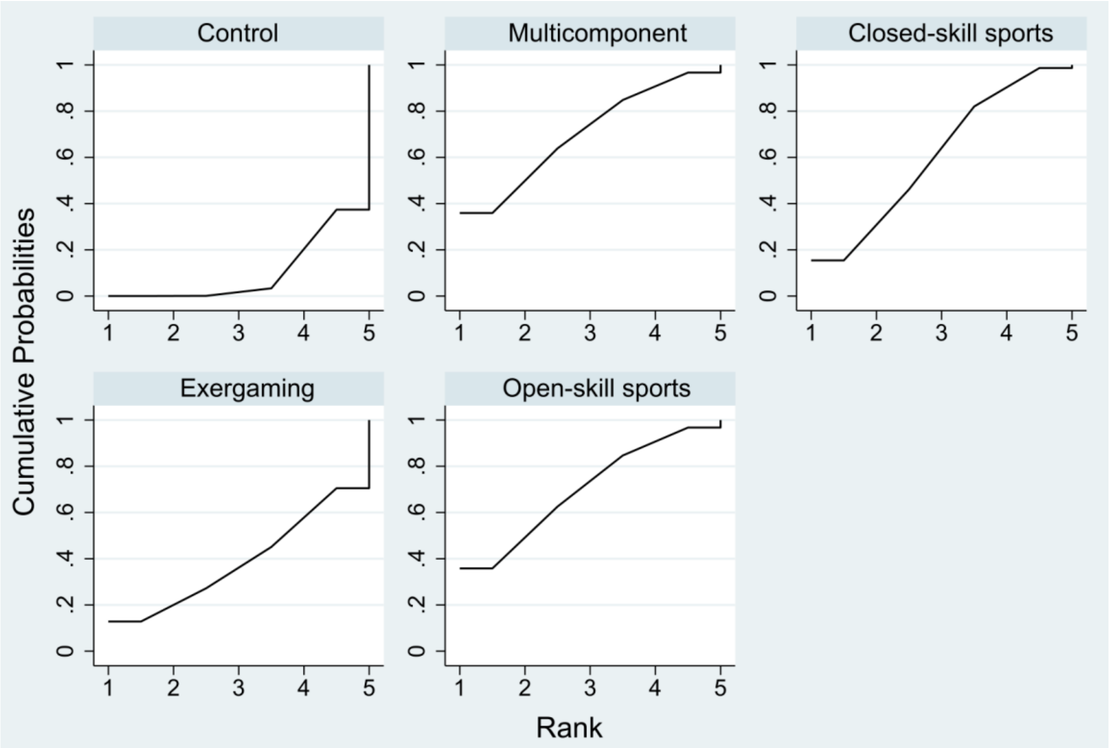

Plots of the surface under the cumulative ranking curves (SUCRA) for all comparisons in the cognitive flexibility.

Figure S13 Relative effect sizes of treatments efficacy based on network meta-analysis for cognitive flexibility

High probability of being the best treatment (High SUCRA values)

**Multicomponent PA**				
0.01 (-2.33 to 2.34)	**Open-skill activities**			
0.30 (-1.64 to 2.24)	0.29 (-1.68 to 2.26)	**Closed-skill activities**		
0.87 (-1.89 to 3.62)	0.86 (-1.92 to 3.64)	0.57 (-1.89 to 3.02)	**Exergaming**	
1.44 (-0.19 to 3.07)	1.43 (-0.24 to 3.11)	1.14 (0.09 to 2.20)	0.58 (-1.64 to 2.79)	**Control**

High probability of being the worst treatment (Low SUCRA values)

S8. Publication bias assessed with funnel plots for executive functions

Figure S14 Comparison-adjusted funnel plot for the inhibitory control network

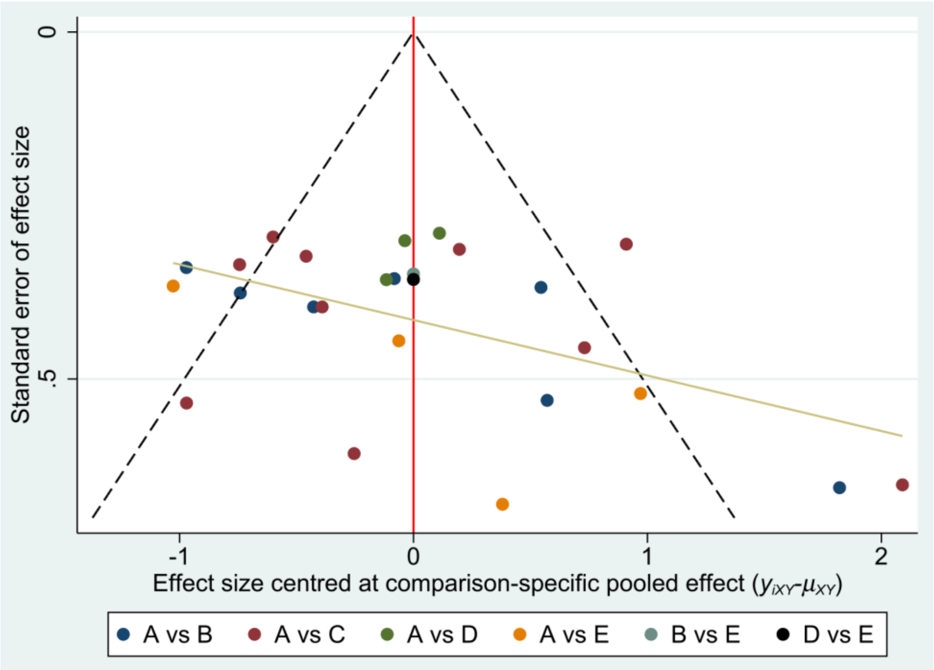

Figure S15 Comparison-adjusted funnel plot for the working memory network

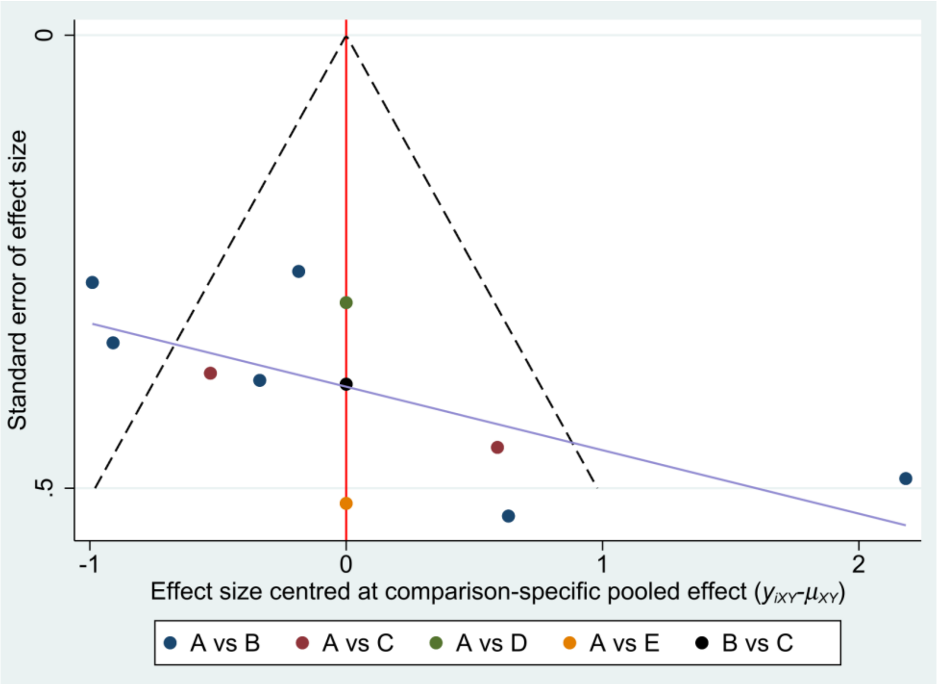

Figure S16 Comparison-adjusted funnel plot for the cognitive flexibility network

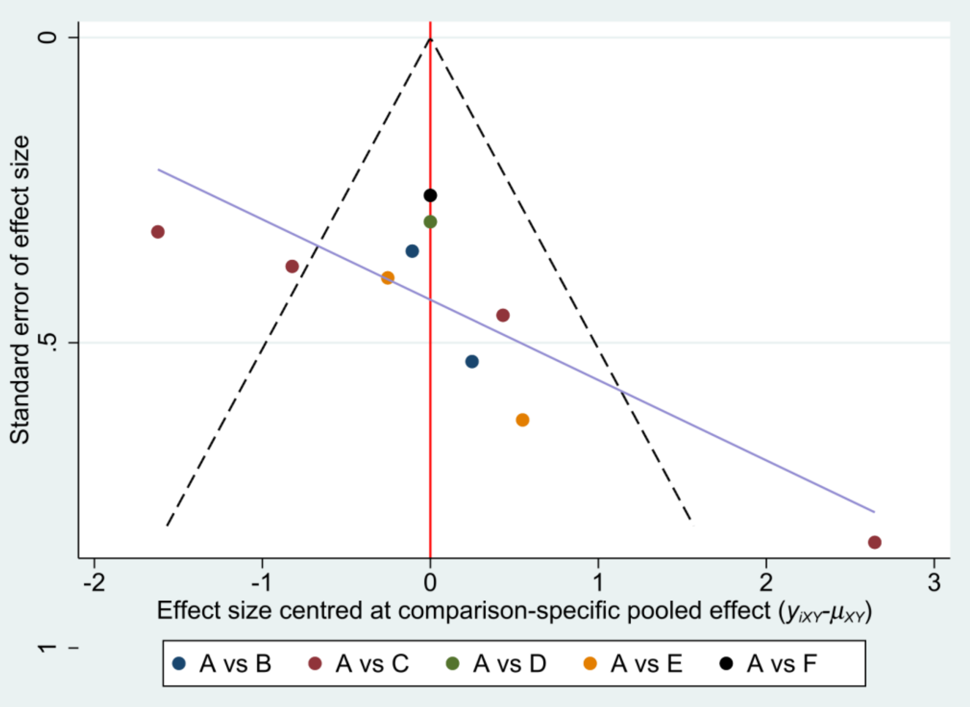

S9. Meta-regression analyses for executive functions

Figure S17 The results of meta-regression analyses for executive functions

```
1. Empirical mean and standard deviation for each variable,
   plus standard error of the mean:

             Mean     SD Naive SE Time-series SE
d.1.2      -5.486  33.26  0.07438        0.05904
d.1.3      -7.474  45.89  0.10261        0.07430
d.1.4      53.634  46.24  0.10339        0.21210
d.1.5       9.466  36.17  0.08089        0.24116
sd.d       72.784  14.20  0.03175        0.07296
beta[2]    61.152 147.28  0.32932        2.68689
beta[3]   -52.624 173.51  0.38797        2.48008
beta[4]   -46.523  90.23  0.20175        1.67674
beta[5]    22.289  40.41  0.09036        4.79158

2. Quantiles for each variable:

            2.5%      25%     50%    75%  97.5%
d.1.2     -65.21  -30.814  -7.270  20.44  56.64
d.1.3     -98.84  -40.537  -2.725  29.49  65.69
d.1.4     -37.48   23.302  53.691  83.77 145.33
d.1.5     -61.68  -14.519   9.587  33.49  80.22
sd.d       49.76   62.281  70.982  81.75 104.89
beta[2]  -177.90  -45.205  95.149 197.73 219.81
beta[3]  -303.22 -188.890 -29.329  95.77 153.45
beta[4]  -236.99 -101.322 -42.237  11.40 124.19
beta[5]   -58.43   -3.595  21.564  48.67 105.27

-- Model fit (residual deviance):

    Dbar        pD       DIC
 59.68413  59.57809 119.26222

60 data points, ratio 0.9947, I^2 = 1%
```

Frequency

```
1. Empirical mean and standard deviation for each variable,
   plus standard error of the mean:

              Mean     SD Naive SE Time-series SE
d.1.2        3.195  31.97  0.07149        0.11707
d.1.3       10.426  29.27  0.06545        0.06062
d.1.4       63.410  62.45  0.13965        0.83461
d.1.5       15.209  55.19  0.12341        2.51334
sd.d        92.756  28.64  0.06404        0.33395
beta[2]   -134.064 267.77  0.59875        1.93663
beta[3]     75.541 196.18  0.43866       10.31818
beta[4]     53.412 111.77  0.24992        5.12928
beta[5]      3.580  97.12  0.21717       27.90645

2. Quantiles for each variable:

            2.5%      25%      50%     75%  97.5%
d.1.2     -61.63  -15.889    3.581  21.574  69.81
d.1.3     -48.36   -6.985   10.090  27.180  72.30
d.1.4     -62.75   24.472   63.320 102.297 190.20
d.1.5     -96.90  -17.612   15.501  47.220 130.31
sd.d       33.73   76.440  107.970 114.201 116.78
beta[2]  -411.45 -335.930 -211.048  -6.783 304.15
beta[3]  -246.20  -32.453  100.148 174.012 373.85
beta[4]  -162.82  -14.318   49.465 117.996 284.31
beta[5]  -191.80  -55.466    2.781  50.791 234.38

-- Model fit (residual deviance):

    Dbar        pD       DIC
 60.12699  59.88563 120.01263

60 data points, ratio 1.002, I^2 = 2%
```

Intensity

```
1. Empirical mean and standard deviation for each variable,
   plus standard error of the mean:

              Mean     SD Naive SE Time-series SE
d.1.2       -2.721  41.73  0.09330        0.07622
d.1.3       -2.024  27.14  0.06068        0.05953
d.1.4       40.738  59.56  0.13317        0.42610
d.1.5      -10.005  62.37  0.13946        1.78242
sd.d        89.197  33.67  0.07530        0.06562
beta[2]      5.699 318.68  0.71259        1.77069
beta[3]   -170.018 110.94  0.24807        3.23154
beta[4]    -58.316 100.38  0.22445        1.96895
beta[5]     94.306 160.01  0.35778       29.53184

2. Quantiles for each variable:

            2.5%      25%      50%      75%  97.5%
d.1.2     -98.35  -25.333    5.762   22.180  70.03
d.1.3     -60.51  -18.640    1.577   14.638  49.71
d.1.4     -80.66    6.786   38.519   74.573 166.15
d.1.5    -151.59  -48.583    2.016   33.756  90.75
sd.d       25.62   67.176  106.098  114.188 116.81
beta[2]  -301.75 -184.538 -111.585   87.320 543.93
beta[3]  -292.94 -242.965 -203.325 -122.932  18.37
beta[4]  -271.10 -116.669  -55.308    3.941 135.93
beta[5]  -141.73  -21.680   52.870  184.445 453.49

-- Model fit (residual deviance):

    Dbar        pD       DIC
 59.32767  59.26679 118.59446

60 data points, ratio 0.9888, I^2 = 0.6%
```

Duration

```
1. Empirical mean and standard deviation for each variable,
   plus standard error of the mean:

              Mean      SD Naive SE Time-series SE
d.1.2        4.142  15.081  0.03372         0.1324
d.1.3      -37.369  17.006  0.03803         0.1546
d.1.4       31.759  31.463  0.07035         0.1480
d.1.5       14.099  22.664  0.05068         0.3777
sd.d        44.525   9.132  0.02042         0.1091
beta[2]   -210.405 153.631  0.34353         4.9610
beta[3]   -276.574  70.211  0.15700         6.5498
beta[4]   -126.473 155.298  0.34726         1.5388
beta[5]   -202.883 152.215  0.34036         1.4528

2. Quantiles for each variable:

            2.5%       25%      50%     75%    97.5%
d.1.2     -25.70   -5.6254    4.112   13.82   34.287
d.1.3     -74.11  -47.9211  -36.045  -25.53   -7.227
d.1.4     -29.45   11.1038   31.356   52.05   95.110
d.1.5     -30.69   -0.4763   14.030   28.55   59.342
sd.d       29.94   37.8359   43.479   50.08   64.947
beta[2]  -426.93 -343.7890 -212.081  -86.22   11.372
beta[3]  -404.87 -318.6580 -266.985 -215.57 -171.765
beta[4]  -488.60 -213.9582 -101.443  -19.37  122.065
beta[5]  -424.53 -339.7806 -211.227  -60.94   27.259

-- Model fit (residual deviance):

    Dbar        pD       DIC
 59.29099  58.70358 117.99458

60 data points, ratio 0.9882, I^2 = 0.5%
```

Length

S10. GRADE assessment for executive functions

Table S7 Summary of GRADE assessment for the certainty in executive functions

Comparisons	Nature of evidence	Certainty	Reason for downgrading
Open-skill sports vs. closed-skill sports	Indirect	Very low	Imprecision, indirectness
Open-skill sports vs. multicomponent	Mixed	Very low	Imprecision, inconsistency
Open-skill sports vs. exergaming	Mixed	Moderate	Risk of bias
Open-skill sports vs. control	Mixed	High	No downgrade
Closed-skill sports vs. multicomponent	Mixed	Low	Imprecision
Closed-skill sports vs. exergaming	Indirect	Low	Imprecision, indirectness
Closed-skill sports vs. control	Mixed	Moderate	Risk of bias
Multicomponent vs. exergaming	Indirect	Low	Imprecision, risk of bias
Multicomponent vs. control	Mixed	Moderate	Risk of bias
Exergaming vs. control	Mixed	Low	Imprecision, risk of bias

S11. Analysis for hyperactivity/impulsivity

Figure S18 Loop-specific heterogeneity for hyperactivity/impulsivity

Loop	IF	seIF	z_value	p_value	CI_95	Loop_Heterog_tau2
A-B-F	1.312	5.464	0.240	0.810	(0.00,12.02)	4.098

Inconsistency plot for the hyperactivity/impulsivity, assuming loop-specific heterogeneity estimates.

Figure S19 The ranking of effects for hyperactivity/impulsivity

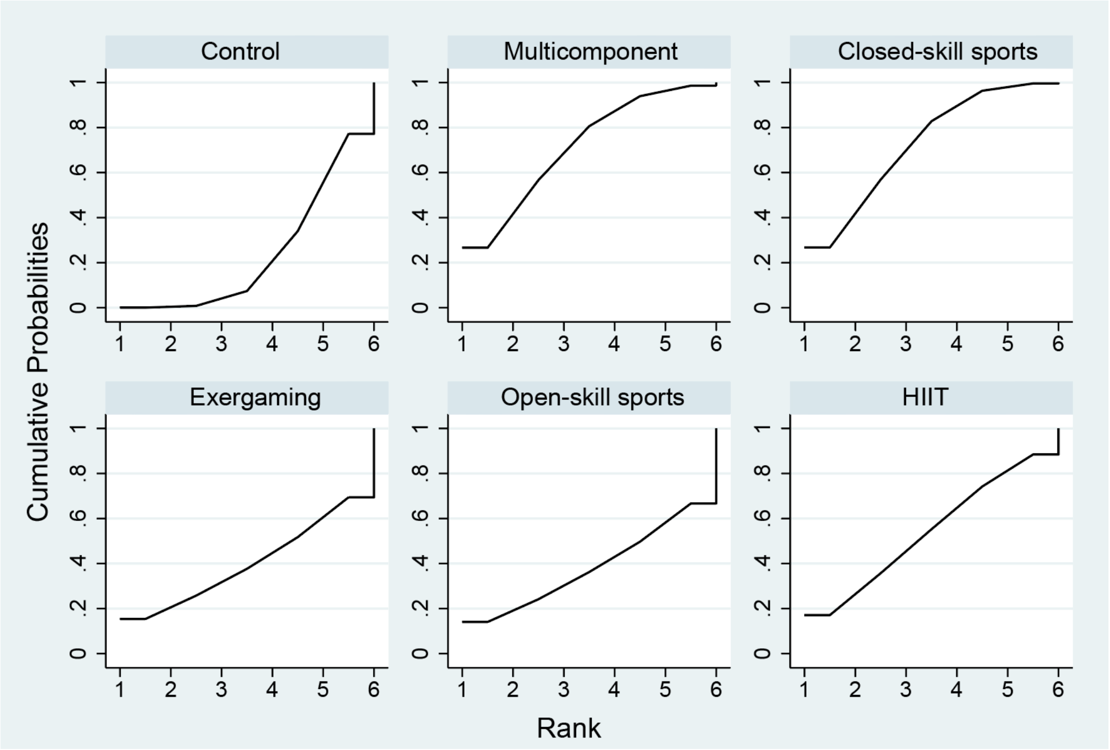

Plots of the surface under the cumulative ranking curves (SUCRA) for all comparisons in the hyperactivity/impulsivity network.

S12. Analysis for inattention

Figure S20 Loop-specific heterogeneity for inattention

Loop	IF	seIF	z_value	p_value	CI_95	Loop_Heterog_tau2
A-B-F	0.358	0.512	0.700	0.484	(0.00,1.36)	0.000

Inconsistency plot for the inattention, assuming loop-specific heterogeneity estimates.

Figure S21 The ranking of effects for inattention

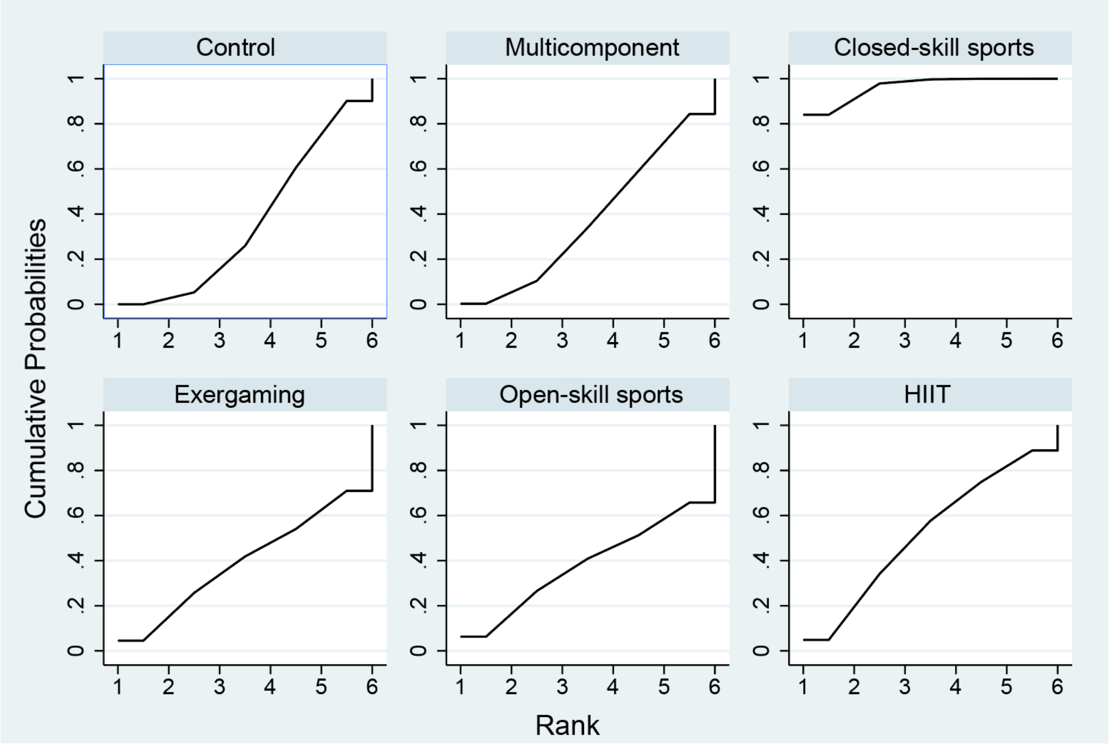

Plots of the surface under the cumulative ranking curves (SUCRA) for all comparisons in the inattention network.

S13. Publication bias assessed with funnel plots for major symptoms

Figure S22 Comparison-adjusted funnel plot for the hyperactivity/impulsivity network

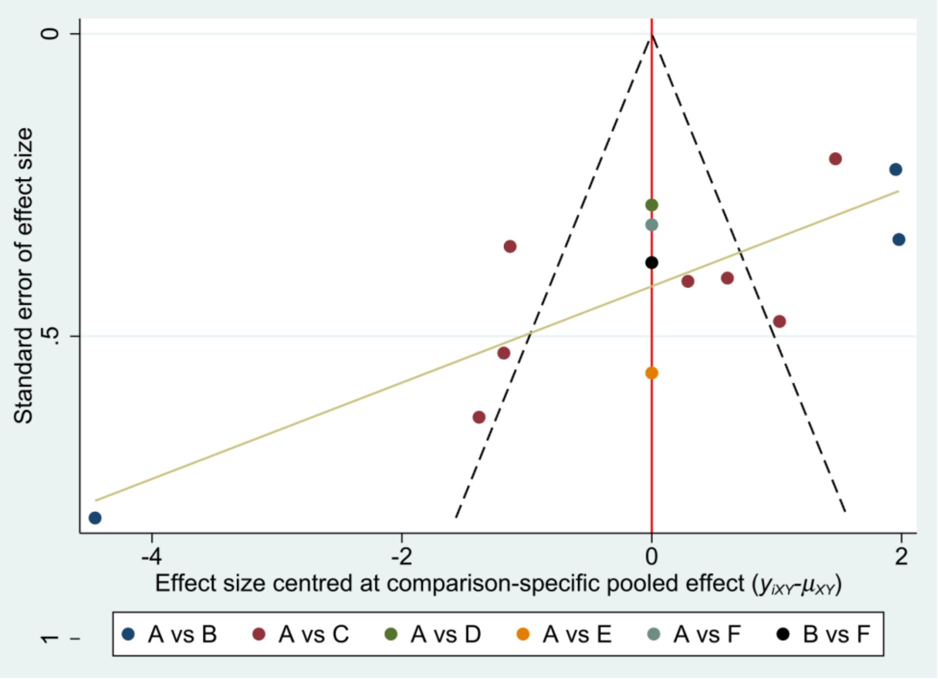

Figure S23 Comparison-adjusted funnel plot for the inattention network

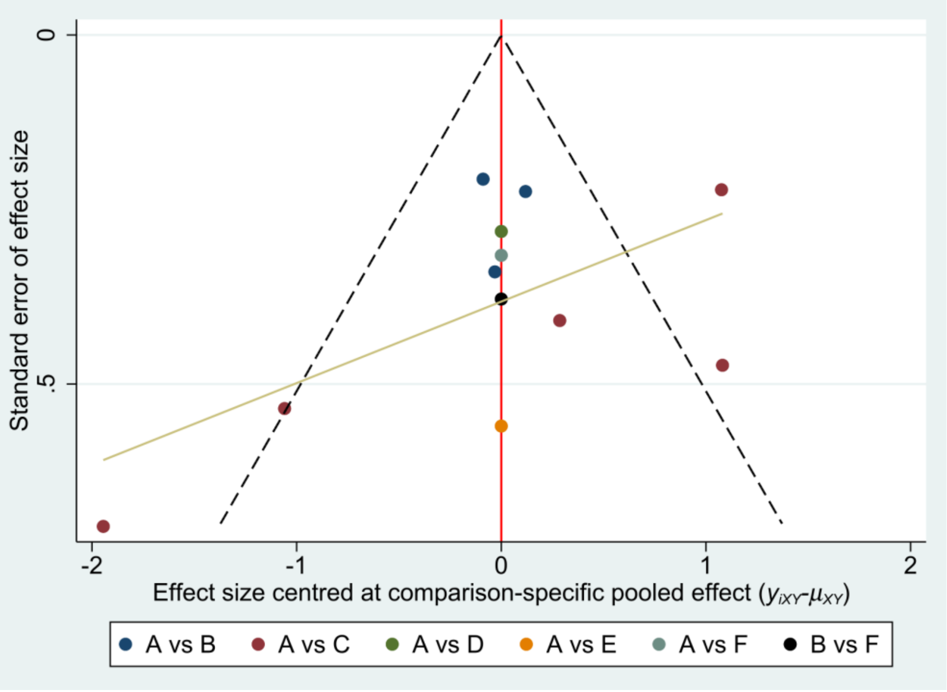

S14. Meta-regression analyses for major symptoms

Figure S24 The results of meta-regression analyses for hyperactivity/impulsivity

```
1. Empirical mean and standard deviation for each variable,
   plus standard error of the mean:

             Mean      SD Naive SE Time-series SE
d.1.2     -0.5812   3.085 0.006899       0.021236
d.1.3     -5.4553   2.195 0.004908       0.006217
d.1.4     -1.5094   6.531 0.014604       0.103103
d.1.5     -2.9040  26.179 0.058538       0.995287
d.1.6     -1.6540   4.881 0.010915       0.084347
sd.d       5.1460   1.867 0.004174       0.026200
beta[2]    1.2361   6.488 0.014509       1.132292
beta[3]    6.4383   3.392 0.007585       0.576225
beta[4]   -0.7476 139.261 0.311397       4.799500
beta[5]    1.7722  81.373 0.181955       3.398466
beta[6]   -8.4963  93.248 0.208509       4.991421

2. Quantiles for each variable:

              2.5%     25%      50%    75%   97.5%
d.1.2      -6.8293  -2.399 -0.56917  1.249   5.617
d.1.3      -9.9394  -6.754 -5.41262 -4.132  -1.125
d.1.4     -14.2462  -5.172 -1.52422  2.129  11.613
d.1.5     -52.2607 -12.981 -3.38063  6.152  55.786
d.1.6     -11.4081  -4.590 -1.69322  1.217   8.459
sd.d        2.5541   3.845  4.79175  6.054   9.786
beta[2]    -9.5725  -3.685  0.75760  5.165  15.634
beta[3]     0.6883   4.027  6.09224  8.475  14.021
beta[4]  -228.5349 -19.038 -0.11935 18.566 212.332
beta[5]  -154.3489 -17.769  0.04431 17.965 191.149
beta[6]  -267.7240 -21.814 -1.17367 17.775 170.818

-- Model fit (residual deviance):

    Dbar       pD      DIC
28.25290 27.27184 55.52474

28 data points, ratio 1.009, I^2 = 4%
```

Frequency

```
1. Empirical mean and standard deviation for each variable,
   plus standard error of the mean:

              Mean      SD Naive SE Time-series SE
d.1.2      -0.7493   3.604 0.008059        0.04618
d.1.3      -4.5065   2.610 0.005835        0.03069
d.1.4      -3.8149  27.144 0.060695        2.68218
d.1.5      -3.2766  40.382 0.090298        2.07924
d.1.6      12.8579  29.130 0.065137        2.12090
sd.d        5.7165   2.323 0.005194        0.05207
beta[2]     1.5202   6.144 0.013739        2.05077
beta[3]     4.5842   5.407 0.012091        0.60388
beta[4]    -4.7028  54.476 0.121812        5.60773
beta[5]    -0.2878  60.649 0.135616        3.13102
beta[6]   138.4542 265.517 0.593713       24.50683

2. Quantiles for each variable:

             2.5%     25%     50%     75%   97.5%
d.1.2      -8.686  -2.655 -0.6072   1.362  6.0932
d.1.3     -10.130  -5.987 -4.3506  -2.880  0.3141
d.1.4     -60.461 -11.926 -2.1137   7.443 44.4534
d.1.5     -97.842 -17.881 -3.1702  11.867 86.9248
d.1.6     -18.794  -4.736  0.2603  18.829 84.7473
sd.d        2.605   4.108  5.2322   6.777 11.7273
beta[2]    -7.036  -1.916  0.6971   3.153 21.6595
beta[3]    -6.975   1.136  4.9575   8.309 14.5036
beta[4]  -119.848 -17.867 -1.1542  15.033 92.7916
beta[5]  -137.792 -18.745 -0.3204  17.537 144.8557
beta[6]  -117.531 -11.539  8.3265 166.304 799.2862

-- Model fit (residual deviance):

    Dbar       pD      DIC
29.02003 27.65391 56.67395

28 data points, ratio 1.036, I^2 = 7%
```

Intensity

```
1. Empirical mean and standard deviation for each variable,
   plus standard error of the mean:

             Mean      SD Naive SE Time-series SE
d.1.2     -0.3208   4.304 0.009624        0.07900
d.1.3     -5.5271   2.927 0.006545        0.02329
d.1.4     -3.5194  18.108 0.040491        1.18002
d.1.5     -8.3134  49.515 0.110718        6.31522
d.1.6     -2.0667   6.109 0.013661        0.09206
sd.d       6.8054   2.450 0.005478        0.04633
beta[2]   -0.5968   9.684 0.021653        1.56276
beta[3]    1.3807   4.654 0.010407        0.63109
beta[4]   -6.7224  56.938 0.127317        3.82885
beta[5]  -16.8375 166.351 0.371971       20.63453
beta[6]    2.2116  13.752 0.030751        1.23620

2. Quantiles for each variable:

            2.5%     25%      50%    75%    97.5%
d.1.2     -8.904  -2.912 -0.34354  2.229   8.4657
d.1.3    -11.596  -7.235 -5.45416 -3.754   0.2086
d.1.4    -52.336  -9.857 -1.89190  5.728  26.5852
d.1.5    -85.090 -13.702 -3.42559  6.815  48.9464
d.1.6    -14.154  -5.767 -2.10760  1.511  10.5028
sd.d       3.445   5.076  6.31948  7.997  13.0743
beta[2]  -18.602  -6.589 -1.11532  4.731  22.1422
beta[3]   -8.457  -1.380  1.09995  4.227  10.9737
beta[4] -170.847 -18.882 -0.57326 16.532  85.2987
beta[5] -275.011 -18.383  0.08046 18.614 173.8948
beta[6]  -23.956  -6.256  1.57100 10.131  30.8826

-- Model fit (residual deviance):

    Dbar       pD      DIC
28.20935 27.55312 55.76247

28 data points, ratio 1.007, I^2 = 4%
```

Duration

```
1. Empirical mean and standard deviation for each variable,
   plus standard error of the mean:

             Mean     SD Naive SE Time-series SE
d.1.2      0.5798  6.221 0.013911        0.07922
d.1.3     -4.8710  3.162 0.007071        0.01593
d.1.4     -3.9461 19.878 0.044449        1.73929
d.1.5     -3.5579 15.496 0.034649        0.27860
d.1.6     -2.2788 16.306 0.036462        2.40598
sd.d       7.3199  3.136 0.007013        0.09077
beta[2]   -2.6801 12.542 0.028046        1.93115
beta[3]    6.0030  6.467 0.014460        0.37275
beta[4]  -11.0508 83.599 0.186932        7.98800
beta[5]   -0.0343 98.868 0.221077        2.68488
beta[6]   -3.5561 32.833 0.073416        6.70267

2. Quantiles for each variable:

            2.5%     25%       50%    75%   97.5%
d.1.2    -12.642  -2.990  1.090387  4.403  12.437
d.1.3    -11.664  -6.625 -4.711963 -3.006   1.248
d.1.4    -49.308  -9.394 -2.052721  4.820  26.723
d.1.5    -32.686 -11.662 -3.496361  4.722  25.820
d.1.6    -36.338 -12.390 -2.431034  7.558  31.105
sd.d       2.845   4.995  6.741931  9.059  15.065
beta[2]  -23.372 -11.841 -4.044479  4.519  19.939
beta[3]   -7.108   1.855  6.277181 10.261  18.467
beta[4] -211.099 -19.997 -1.247831 15.342 109.021
beta[5] -183.795 -18.786  0.003472 18.632 176.454
beta[6]  -69.979 -23.828 -3.898496 13.285  67.490

-- Model fit (residual deviance):

    Dbar       pD      DIC
28.53632 27.84102 56.37734

28 data points, ratio 1.019, I^2 = 5%
```

Length

Figure S25 The results of meta-regression analyses for inattention

```
1. Empirical mean and standard deviation for each variable,
   plus standard error of the mean:

              Mean      SD Naive SE Time-series SE
d.1.2    -765.7497  322.75   0.7217         0.5181
d.1.3     203.0258  207.01   0.4629         1.2455
d.1.4       0.8808  384.68   0.8602         1.0512
d.1.5     175.6291  384.45   0.8597         1.5661
d.1.6    -331.2501  304.22   0.6803         0.7678
sd.d      381.5777   53.02   0.1186         0.2129
beta[2]  1375.8814  448.10   1.0020         3.1573
beta[3]   830.3693  460.35   1.0294        45.8009
beta[4]   -55.5421  698.51   1.5619        71.9162
beta[5]  -102.8309  680.50   1.5216        37.4808
beta[6]  -460.6770 1615.18   3.6116       111.4816

2. Quantiles for each variable:

           2.5%     25%       50%    75%  97.5%
d.1.2   -1335.8 -1005.4 -796.2192 -519.4 -147.5
d.1.3    -203.8    65.5  203.1539  338.5  615.2
d.1.4    -762.2  -252.2    0.9565  254.4  763.0
d.1.5    -501.4   -93.7  134.8288  426.4  985.9
d.1.6    -949.2  -529.6 -322.8615 -126.2  249.0
sd.d      238.8   360.9  399.2422  420.7  435.0
beta[2]   650.0  1161.0 1517.6509 1741.3 1810.6
beta[3]   128.8   453.2  761.3566 1169.4 1698.0
beta[4] -1774.5  -326.8  -12.0002  274.6 1220.2
beta[5]  -864.3  -663.3 -322.2507  305.4 1192.8
beta[6] -3464.2 -1242.0 -172.9161  896.6 1667.1

-- Model fit (residual deviance):

    Dbar       pD      DIC
26.01698 26.01658 52.03356

26 data points, ratio 1.001, I^2 = 4%
```

Frequency

```
1. Empirical mean and standard deviation for each variable,
   plus standard error of the mean:

            Mean     SD Naive SE Time-series SE
d.1.2    132.762  209.6   0.4688         0.4453
d.1.3    -12.647  144.6   0.3234         0.3232
d.1.4     -2.276  348.5   0.7792         5.6318
d.1.5    275.541  236.0   0.5278         1.2007
d.1.6    227.596  894.0   1.9990         0.7175
sd.d     293.973  132.6   0.2964         1.1397
beta[2] -668.542  661.5   1.4792         8.3149
beta[3] -367.752  325.7   0.7283        69.7450
beta[4]  -18.942 1130.5   2.5279       285.7163
beta[5] -674.084  692.6   1.5487       101.8300
beta[6] -104.112 1549.4   3.4646         5.4004

2. Quantiles for each variable:

            2.5%      25%      50%     75%   97.5%
d.1.2    -311.35    23.88  134.828  248.53  559.21
d.1.3    -321.11   -86.17   -9.645   60.41  291.24
d.1.4    -711.73  -197.28  -16.957  190.93  735.67
d.1.5    -220.59   150.49  282.475  392.60  777.05
d.1.6    -641.77  -420.26 -171.748  771.00 2088.87
sd.d       51.48   184.49  349.846  401.60  433.28
beta[2] -1399.47 -1268.76 -804.363 -174.30  295.60
beta[3] -1036.76  -631.15 -284.033  -85.29   82.78
beta[4] -1743.34  -609.39  -37.844  316.03 3782.31
beta[5] -2168.57 -1045.81 -620.733 -124.82  424.51
beta[6] -1349.65 -1315.02 -785.369  446.97 2492.09

-- Model fit (residual deviance):

    Dbar       pD      DIC
25.99697 25.99628 51.99325

26 data points, ratio 0.9999, I^2 = 4%
```

Intensity

```
1. Empirical mean and standard deviation for each variable,
   plus standard error of the mean:

            Mean      SD Naive SE Time-series SE
d.1.2    -26.842  169.88   0.3799         0.4086
d.1.3   -162.220  167.77   0.3751         0.7733
d.1.4    -55.765  525.68   1.1755         8.5620
d.1.5    439.904  311.04   0.6955         4.3656
d.1.6      1.387  225.76   0.5048         0.5456
sd.d     262.084   90.78   0.2030         0.8559
beta[2]   20.985  349.57   0.7817        10.4803
beta[3]  408.895  299.34   0.6693        17.1421
beta[4] -220.677 1784.66   3.9906       194.7646
beta[5]  455.421  496.60   1.1104        47.9472
beta[6] -369.315  353.20   0.7898        15.4733

2. Quantiles for each variable:

           2.5%     25%      50%    75%  97.5%
d.1.2    -353.3 -143.75  -24.250  84.83  310.3
d.1.3    -563.0 -246.13 -133.269 -52.17  109.0
d.1.4   -1186.2 -332.20    6.694 253.91  935.2
d.1.5    -121.1  201.42  450.003 666.08 1029.2
d.1.6    -433.6 -145.70   -4.027 140.90  468.0
sd.d      115.0  187.13  251.741 338.35  425.5
beta[2]  -583.3  -66.85  132.169 262.04  370.7
beta[3]   115.0  199.17  259.590 548.78  960.3
beta[4] -4239.0 -924.02  118.404 852.48 2289.1
beta[5]  -448.3  -11.21  647.521 871.86 1056.2
beta[6]  -874.9 -671.06 -354.544 -97.13  191.0

-- Model fit (residual deviance):

    Dbar       pD      DIC
25.99193 25.99003 51.98196

26 data points, ratio 0.9997, I^2 = 4%
```

Duration


```
1. Empirical mean and standard deviation for each variable,
   plus standard error of the mean:

            Mean     SD Naive SE Time-series SE
d.1.2    132.762  209.6   0.4688         0.4453
d.1.3    -12.647  144.6   0.3234         0.3232
d.1.4     -2.276  348.5   0.7792         5.6318
d.1.5    275.541  236.0   0.5278         1.2007
d.1.6    227.596  894.0   1.9990         0.7175
sd.d     293.973  132.6   0.2964         1.1397
beta[2] -668.542  661.5   1.4792         8.3149
beta[3] -367.752  325.7   0.7283        69.7450
beta[4]  -18.942 1130.5   2.5279       285.7163
beta[5] -674.084  692.6   1.5487       101.8300
beta[6] -104.112 1549.4   3.4646         5.4004

2. Quantiles for each variable:

            2.5%      25%      50%     75%   97.5%
d.1.2    -311.35    23.88  134.828  248.53  559.21
d.1.3    -321.11   -86.17   -9.645   60.41  291.24
d.1.4    -711.73  -197.28  -16.957  190.93  735.67
d.1.5    -220.59   150.49  282.475  392.60  777.05
d.1.6    -641.77  -420.26 -171.748  771.00 2088.87
sd.d       51.48   184.49  349.846  401.60  433.28
beta[2] -1399.47 -1268.76 -804.363 -174.30  295.60
beta[3] -1036.76  -631.15 -284.033  -85.29   82.78
beta[4] -1743.34  -609.39  -37.844  316.03 3782.31
beta[5] -2168.57 -1045.81 -620.733 -124.82  424.51
beta[6] -1349.65 -1315.02 -785.369  446.97 2492.09

-- Model fit (residual deviance):

    Dbar       pD      DIC
25.99697 25.99628 51.99325

26 data points, ratio 0.9999, I^2 = 4%
```


Length

S15. GRADE assessment for major symptoms

Table S8 Summary of GRADE assessment for the certainty in hyperactivity/ impulsivity

Comparisons	Nature of evidence	Certainty	Reason for downgrading
Closed-skill sports vs. multicomponent	Indirect	Low	Imprecision, indirectness
Closed-skill sports vs. HIIT	Indirect	Low	Imprecision, indirectness
Closed-skill sports vs. exergaming	Indirect	Low	Imprecision, indirectness
Closed-skill sports vs. open-skill sports	Indirect	Low	Imprecision, indirectness
Closed-skill sports vs. control	Mixed	Moderate	Risk of bias
Multicomponent vs. HIIT	Mixed	Low	Imprecision
Multicomponent vs. exergaming	Indirect	Low	Imprecision, indirectness
Multicomponent vs. open-skill sports	Indirect	Low	Imprecision, indirectness
Multicomponent vs. control	Mixed	Low	Imprecision, inconsistency
HIIT vs. exergaming	Indirect	Low	Imprecision, indirectness
HIIT vs. open-skill sports	Indirect	Low	Imprecision, indirectness
HIIT vs. control	Mixed	Very low	Imprecision, inconsistency
Exergaming vs. open-skill sports	Indirect	Low	Imprecision, indirectness
Exergaming vs. control	Mixed	Low	Imprecision, risk of bias
Open-skill sports vs. control	Mixed	Very low	Imprecision, inconsistency

Table S9 Summary of GRADE assessment for the certainty in inattention

Comparisons	Nature of evidence	Certainty	Reason for downgrading
Closed-skill sports vs. HIIT	Indirect	Low	Imprecision, indirectness
Closed-skill sports vs. exergaming	Indirect	Low	Imprecision, indirectness
Closed-skill sports vs. open-skill sports	Indirect	Low	Imprecision, indirectness
Closed-skill sports vs. multicomponent	Indirect	Very low	Imprecision, indirectness
Closed-skill sports vs. control	Mixed	High	No downgrade
HIIT vs. exergaming	Indirect	Low	Imprecision, indirectness
HIIT vs. open-skill sports	Indirect	Low	Imprecision, indirectness
HIIT vs. multicomponent	Mixed	Low	Imprecision
HIIT vs. control	Mixed	Low	Imprecision, risk of bias
Exergaming vs. open-skill sports	Indirect	Low	Imprecision, indirectness
Exergaming vs. multicomponent	Indirect	Low	Imprecision, indirectness
Exergaming vs. control	Mixed	Low	Imprecision, risk of bias
Open-skill sports vs. multicomponent	Indirect	Low	Imprecision, indirectness
Open-skill sports vs. control	Mixed	Very low	Imprecision, inconsistency
Multicomponent vs. control	Mixed	Low	Imprecision, risk of bias

S16. A partially contextualized framework

Table S10 A partially contextualized framework was used for classification of interventions based on network meta-analysis

Outcomes	Classification	Intervention	Effect size	Surface under the cumulative ranking curve	Certainty of evidence
**Inhibitory Control**	Large beneficial effect	Open-skill activities	1.94 (1.24 to 2.64)	99.1%	High
		Closed-skill activities	1.02 (0.54 to 1.50)	55.2%	Moderate
		Multicomponent PA	1.00 (0.46 to 1.55)	54.4%	Moderate
	Moderate beneficial effect	Exergaming	0.79 (0.00 to 1.59)	40.6%	Low
**Working memory**	Large beneficial effect	Closed-skill activities	1.21 (-0.22 to 2.65)	75.9%	Moderate
		Multicomponent PA	1.05 (0.19 to 1.92)	71.3%	Moderate
	Moderate beneficial effect	Open-skill activities	0.65 (-1.59 to 2.89)	52.2%	Low
	Trivial to no effect	Exergaming	0.00 (-2.08 to 2.08)	29.7%	Very low

Outcomes	Classification	Intervention	Effect size	Surface under the cumulative ranking curve	Certainty of evidence
**Cognitive flexibility**	Large beneficial effect	Multicomponent PA	1.44 (-0.19 to 3.07)	70.3%	Moderate
		Open-skill activities	1.43 (-0.24 to 3.11)	70.0%	Low
		Closed-skill activities	1.14 (0.09 to 2.20)	60.6%	Low
	Moderate beneficial effect	Exergaming	0.58 (-1.64 to 2.79)	38.9%	Low
**Hyperactivity/impulsivity**	Large beneficial effect	Closed-skill activities	-1.60 (-0.32 to -0.19)	72.5%	Moderate
		Multicomponent PA	-1.58 (-3.60 to 0.43)	71.3%	Low
		High-intensity interval training	-0.97 (-3.77 to 1.83)	54.2%	Very low
	Small beneficial effect	Exergaming	-0.28 (-3.96 to 3.40)	40.0%	Low
		Open-skill activities	-0.25 (-4.05 to 3.55)	38.2%	Very low

Outcomes	Classification	Intervention	Effect size	Surface under the cumulative ranking curve	Certainty of evidence
**Inattention**	Large beneficial effect	Closed-skill activities	-1.51 (-2.33 to -0.69)	96.3%	High
	Small beneficial effect	High-intensity interval training	-0.28 (-1.54 to 0.98)	52.1%	Low
	Trivial to no effect	Exergaming	-0.02 (-1.65 to 1.61)	39.4%	Low
		Multicomponent PA	-0.02 (-0.89 to 0.85)	37.6%	Low
	Small harmful effect	Open-skill activities	0.04 (-1.87 to 1.94)	38.1%	Very low
